# CD55 regulates self-renewal and cisplatin resistance in endometrioid tumors

**DOI:** 10.1084/jem.20170438

**Published:** 2017-08-24

**Authors:** Caner Saygin, Andrew Wiechert, Vinay S. Rao, Ravi Alluri, Elizabeth Connor, Praveena S. Thiagarajan, James S. Hale, Yan Li, Anastasia Chumakova, Awad Jarrar, Yvonne Parker, Daniel J. Lindner, Anil Belur Nagaraj, J. Julie Kim, Analisa DiFeo, Fadi W. Abdul-Karim, Chad Michener, Peter G. Rose, Robert DeBernardo, Haider Mahdi, Keith R. McCrae, Feng Lin, Justin D. Lathia, Ofer Reizes

**Affiliations:** 1 Department of Cellular and Molecular Medicine, Lerner Research Institute, Cleveland Clinic, Cleveland, OH; 2 Department of Obstetrics and Gynecology, Women's Health Institute, Cleveland Clinic, Cleveland, OH; 3 Department of Immunology, Lerner Research Institute, Cleveland Clinic, Cleveland, OH; 4 Department of Translational Hematology and Oncology Research, Taussig Cancer Institute, Cleveland Clinic, Cleveland, OH; 5 Pathology and Laboratory Medicine Institute, Department of Anatomical Pathology, Cleveland Clinic, Cleveland, OH; 6 Case Comprehensive Cancer Center, Case Western Reserve University, Cleveland, OH; 7 Department of Molecular Medicine, Cleveland Clinic Lerner College of Medicine, Case Western Reserve University, Cleveland, OH; 8 Department of Obstetrics and Gynecology, Northwestern University, Chicago, IL

## Abstract

Effective targeting of cancer stem cells (CSCs) requires neutralization of self-renewal and chemoresistance, but these phenotypes are often regulated by distinct molecular mechanisms. Here we report the ability to target both of these phenotypes via CD55, an intrinsic cell surface complement inhibitor, which was identified in a comparative analysis between CSCs and non-CSCs in endometrioid cancer models. In this context, CD55 functions in a complement-independent manner and required lipid raft localization for CSC maintenance and cisplatin resistance. CD55 regulated self-renewal and core pluripotency genes via ROR2/JNK signaling and in parallel cisplatin resistance via lymphocyte-specific protein tyrosine kinase (LCK) signaling, which induced DNA repair genes. Targeting LCK signaling via saracatinib, an inhibitor currently undergoing clinical evaluation, sensitized chemoresistant cells to cisplatin. Collectively, our findings identify CD55 as a unique signaling node that drives self-renewal and therapeutic resistance through a bifurcating signaling axis and provides an opportunity to target both signaling pathways in endometrioid tumors.

## Teaser

CD55 is a membrane complement regulatory protein that attenuates complement-mediated cytotoxicity. Saygin et al. elucidate a new role for CD55 as a signaling hub for cancer stem cell self-renewal and cisplatin resistance pathways in endometrioid tumors and open a new line of research into chemotherapeutic-refractory cancers.

## Introduction

Uterine and ovarian cancers are the most common gynecological cancers in the US (Baldwin et al., 2012; Siegel et al., 2016). These tumors are characterized by four main histological subtypes: endometrioid, serous, mucinous, and clear cell carcinoma (Karst and Drapkin, 2010; Kurman and Shih, 2016). Endometrioid carcinomas make up >80% of uterine cancers and contribute to 15% of epithelial ovarian cancers (DiSaia and Creasman, 2012). Endometrioid uterine and ovarian cancers are thought to arise from similar cells of origin (Catasus et al., 2009; Cuellar-Partida et al., 2016). In advanced-stage disease, both uterine and ovarian cancers are treated with cytoreductive surgery and platinum-based cytotoxic chemotherapy (Armstrong et al., 2006). Although many patients achieve clinical remission with this standard approach, advanced-stage uterine and ovarian cancers are prone to recurrence (Hanker et al., 2012). Chemoresistance is generally defined as progression of disease within 6 mo of therapy. Patients with relapsed disease are considered incurable in most cases, and management is intended to prolong life with symptomatic relief (Hanker et al., 2012). Several genomic studies have demonstrated that endometrioid tumors are genetically heterogeneous with diverse molecular subtypes, and an actionable driver gene mutation has not been identified (Cancer Genome Atlas Research Network, 2011, 2013; Tan et al., 2013). Therefore, there is an increasing need to identify pathways driving cisplatin resistance that can be targeted to overcome resistance, which otherwise presents as incurable disease.

Both uterine and ovarian endometrioid tumors are heterogeneous and have been shown to contain a self-renewing cancer stem cell (CSC) population. CSCs are implicated in tumor recurrence and treatment resistance (Kyo et al., 2011; Nagaraj et al., 2015; Wiechert et al., 2016). Endometrioid CSCs can be isolated by well-established surface markers, including CD133, CD44, CD49f, aldehyde dehydrogenase activity, and stem cell reporter systems (Kyo et al., 2011; Wiechert et al., 2016). Using multiple CSC enrichment methods, we identified that decay accelerating factor (CD55) was highly expressed in endometrioid CSCs and cisplatin-resistant cells. CD55 is a glycophosphatidylinositol (GPI)-anchored membrane complement regulatory protein that protects cells from complement-mediated lysis (Lukacik et al., 2004). In ovarian and uterine cancers, CD55 is expressed at higher levels in malignant compared with benign endometrial tissue (Murray et al., 2000; Kapka-Skrzypczak et al., 2015). CD55 expression was also shown to have a prognostic significance in patients with breast cancer (Ikeda et al., 2008). In addition to the canonical effects including the modulation of the efficiency of antitumor mAbs, CD55 has been shown to signal intracellularly and activate receptor tyrosine kinases at lipid rafts (Shenoy-Scaria et al., 1992). The role of noncanonical CD55 signaling in T cell receptor activation has been characterized, but there are limited studies on the intracellular actions of CD55 in cancer (Ventimiglia and Alonso, 2013). On the basis of our initial findings in complement-free conditions, we hypothesized that CD55 may regulate self-renewal and cisplatin resistance in endometrioid tumors through a complement-independent mechanism.

## Results

### CD55 is highly expressed in CSCs and cisplatin-resistant cells

**Figure 1.**
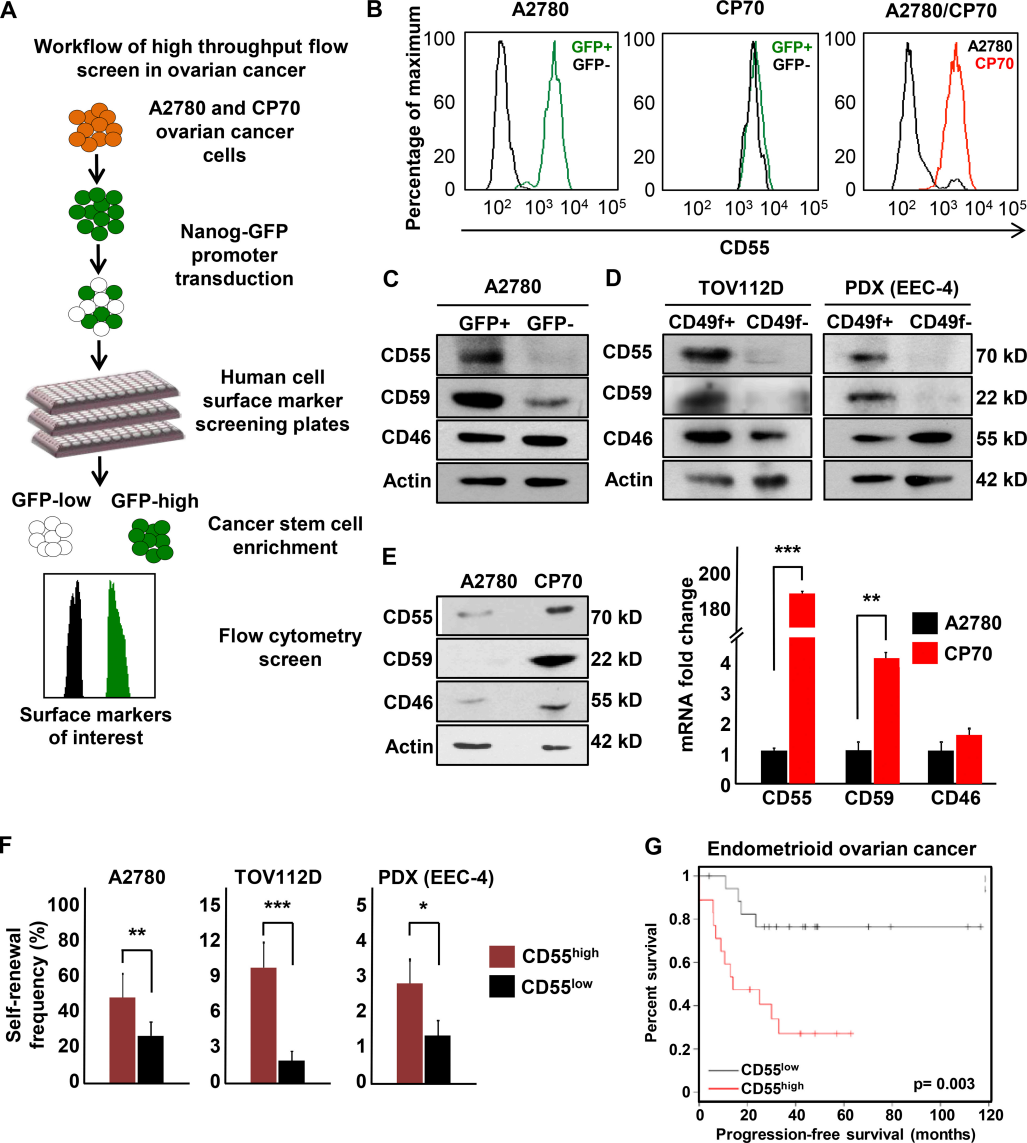
**CD55 is highly expressed on endometrioid ovarian and uterine CSCs and cisplatin-resistant cells.** (A) A high-throughput flow cytometry screen of 242 different surface CD markers in cisplatin-naive (A2780) and cisplatin-resistant (CP70) ovarian cancer cells was performed to investigate the differential expression of these markers between CSCs versus non-CSCs and cisplatin-naive versus cisplatin-resistant cells. (B) Of 242 markers, CD55 was the most highly and differentially expressed between cisplatin-naive CSCs versus non-CSCs and cisplatin-resistant versus cisplatin-naive cells. (C and D) Cell lysates from cisplatin-naive A2780 reporter, TOV112D, and PDX (EEC-4) cells sorted into CSCs and non-CSCs by GFP expression and CD49f expression, respectively, were probed with anti-CD55, CD59, and CD46 antibodies. Actin was used as a loading control. Data are representative of three independent experiments. (E) Protein and mRNA expression of CD55, CD59, and CD46 were assessed in lysates from cisplatin-naive (A2780) and cisplatin-resistant (CP70) cells. Actin was used as a control. Data are representative of two independent experiments. (F) Limiting dilution analysis of CD55+ compared with CD55− cisplatin-naive cells. The graph represents the estimates in percentage of self-renewal frequency in sorted populations with the corresponding p-values. Data represent two independent experiments. (G) Kaplan-Meier (K-M) progression-free survival curve for endometrioid ovarian cancer patients who had high versus low tumor CD55 expression before therapy was obtained from K-M plotter database (http://kmplot.com/analysis/). *, P < 0.05; **, P < 0.01; ***, P < 0.001.

We recently validated the NANOG promoter-driven GFP reporter system in isolation of endometrioid CSCs (Wiechert et al., 2016). We used NANOG-GFP reporter-transduced cisplatin-naive (A2780) and cisplatin-resistant (CP70) ovarian endometrioid tumor cell lines to perform a high-throughput flow cytometry screen (Fig. 1 A). Of 242 cell surface markers included in the screening panel, CD55 was the most differentially expressed protein in between A2780 CSCs (GFP+) and non-CSCs (GFP−; Fig. 1 B). Both GFP+ and GFP− CP70 cells had high levels of CD55 expression, which might be attributed to the higher self-renewal potential and stem-like properties in cisplatin-resistant cells (Wiechert et al., 2016). Of the other two membrane complement regulatory proteins included in the screen, CD59 was expressed higher in CSCs, whereas there was no appreciable difference in CD46 expression (Fig. S1 A). We further validated these results in several cisplatin-naive endometrioid tumor cell lines (A2780, TOV112D) and a patient-derived xenograft model (EEC-4), at the protein and RNA levels (Fig. 1, C and D; and Fig. S1, B–D). Moreover, two primary uterine endometrioid tumor specimen co-expressed CD55 with a CSC marker (UTE-1 and UTE-2; Fig. S1 E). In addition, cisplatin-resistant (CP70) cells had higher expression of CD55 and CD59 at protein and RNA levels, compared with their isogenic cisplatin-naive (A2780) counterparts (Fig. 1 E). CP70 cells had 186- and 4-fold higher expression of CD55 and CD59 mRNA compared with A2780 cells, respectively (Fig. 1 E). We previously reported that CD49f can enrich a self-renewing population in cisplatin-resistant cells (Wiechert et al., 2016). Using this marker, CSCs (CD49f+) isolated from cisplatin-resistant ovarian (CP70) and endometrial (HEC1a) cells had higher levels of CD55 compared with non-CSCs (CD49f−; Fig. S1, F and G). To assess CD55 as a marker of CSCs, we performed limiting dilution sphere formation analysis that provides readout for self-renewal, proliferation, and survival. We found that CD55+ cells isolated from cisplatin-naive (A2780, TOV112D, PDX) and cisplatin-resistant (CP70, HEC1a) endometrioid tumor cells were significantly more self-renewing than their CD55− counterparts (stem cell frequencies for CD55+ vs. CD55− were 1 in 2.2 vs. 1 in 4.3 in A2780 [P < 0.01], 1 in 10.8 vs. 1 in 59.2 in TOV112D [P < 0.001], 1 in 36 vs. 1 in 87.7 in PDX [P < 0.05], 1 in 1.4 vs. 1 in 5.1 in CP70 [P < 0.001], and 1 in 59.6 vs. 1 in 209.7 in HEC1a [P < 0.01]; Fig. 1 F and Fig. S1 H). We next investigated the utility of CD55 in predicting outcomes of patients with endometrioid ovarian cancer by using the Kaplan-Meier plotter biomarker assessment database (Gyorffy et al., 2012). Patients with high tumor CD55 expression at diagnosis had significantly worse progression-free survival compared with patients with low CD55 levels (hazard ratio 4.7, confidence interval 1.5–14.6, P = 0.003; Fig. 1 G). These data demonstrate that CD55 is highly expressed in endometrioid CSCs and cisplatin-resistant cells, enriched in self-renewing populations in both cisplatin-naive and cisplatin-resistant tumors, and predicts survival in patients with endometrioid tumors.

### CD55 is necessary for maintenance of stemness and cisplatin resistance

**Figure 2.**
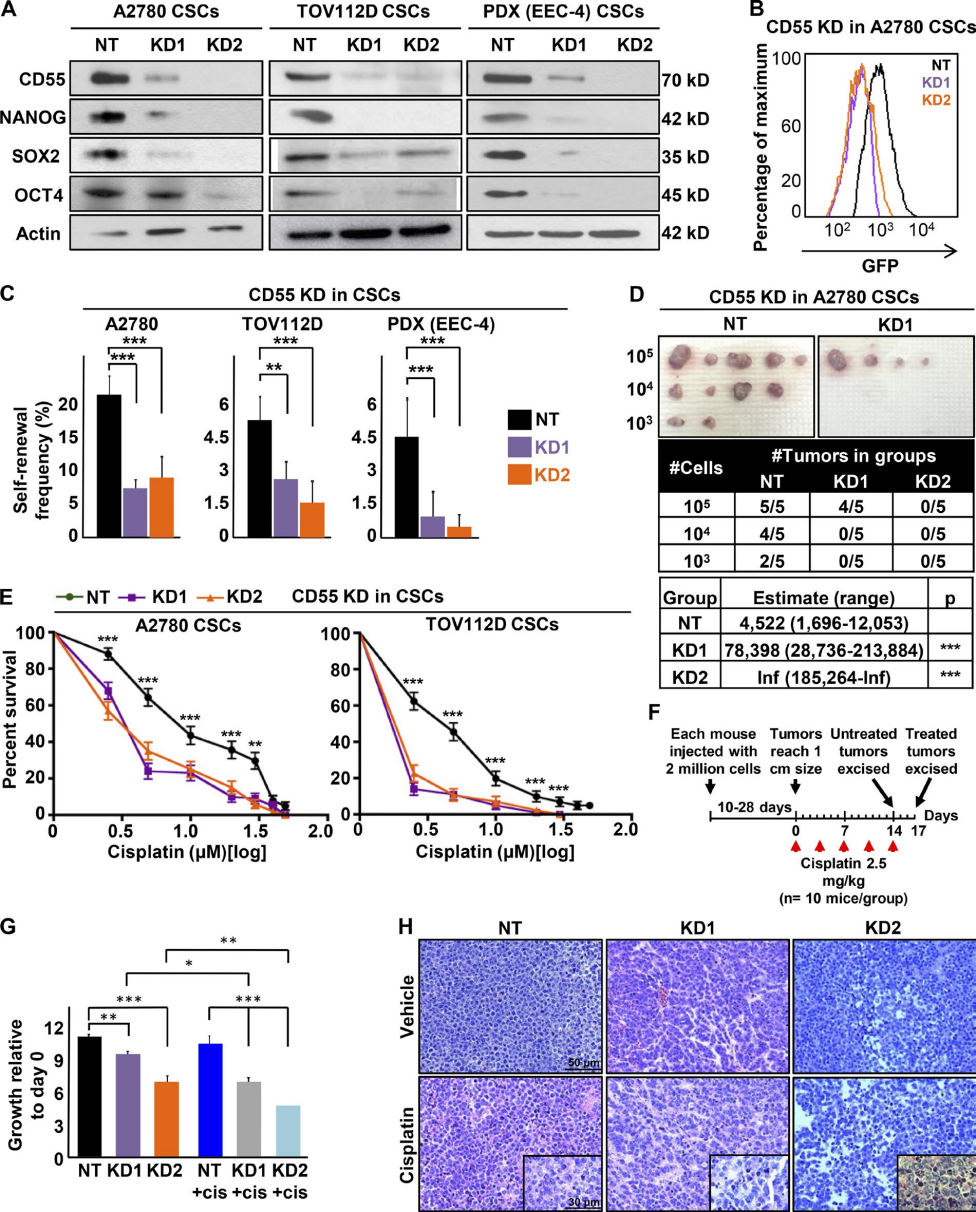
**CD55 maintains self-renewal and cisplatin resistance in endometrioid tumors.** (A) Cell lysates from cisplatin-naive CSCs silenced using two CD55 shRNA constructs (KD1, KD2) and a nontargeting shRNA (NT) control were immunoblotted for CD55, NANOG, SOX2, and OCT4. Actin was used as a loading control. Data are representative of two or three independent experiments. (B) A2780 CSCs silenced for CD55 and NT controls were flowed for GFP signal intensity, which indicates *NANOG* promoter activity. (C) Limiting dilution analysis plots of CD55 NT control compared with CD55 KD1 and KD2 silencing constructs in cisplatin-naive CSCs. (D) In vivo tumor initiation studies were performed with five mice per group, and the estimates of stem cell frequencies of CD55 NT control compared with the CD55 KD1 and KD2 silencing constructs are shown. (E) CD55-silenced cisplatin-naive CSCs and their NT controls were treated with 0–50 µM cisplatin, and percentage surviving cells is graphed. Data are representative of three independent experiments. (F and G) In vivo cisplatin sensitivity studies were performed comparing the NT control group with the CD55-silenced group, and the graph shows the growth rate of tumors compared with the first day of cisplatin treatment. (H) Hematoxylin and eosin–stained slides of tumors excised from mice treated with cisplatin and vehicle controls. *, P < 0.05; **, P < 0.01; ***, P < 0.001. Bar, 50 µm (30 µm in insets).

To investigate functional impact of CD55 in CSCs and cisplatin-resistant cells, we used a genetic approach to inhibit CD55 expression. Using two nonoverlapping CD55 shRNA silencing constructs, we inhibited CD55 mRNA and protein expression in both CSCs and cisplatin-resistant cells (Fig. 2 A and Fig. S2, A–C). No change in expression of CD46 or CD59 was observed (Fig. 2 A and Fig. S2, A–C). Upon CD55 inhibition, the core pluripotency transcription factors' (NANOG, SOX2, and OCT4) expression was inhibited at the RNA and protein levels (Fig. 2 A and Fig. S2, B and C). Concomitantly, we observed a decrease in GFP signal intensity in A2780 CSCs, which indicated decreased NANOG promoter activity (Fig. 2 B). CD55-silenced cisplatin-naive CSC cultures (A2780, TOV112D, PDX) and cisplatin-resistant parental cell cultures (CP70, HEC1a) showed significantly lower self-renewal and stem cell frequencies compared with nontarget control based on limiting dilution sphere formation analysis (nontargeted control to CD55-silenced conditions from 1 in 4.8 to 1 in 14.6 and 1 in 10.6 for A2780 CSCs [P < 0.001]; 1 in 18.6 to 1 in 41.5 [P < 0.01] and 1 in 65 [P < 0.001] for TOV112D CSCs; 1 in 21.9 to 1 in 100 and 1 in 207.1 for PDX CSCs [P < 0.001]; 1 in 3.3 to 1 in 9.6 [P < 0.001] and 1 in 5.9 [P < 0.01] for CP70 parental; and 1 in 22 to 1 in 50.2 [P < 0.01] and 1 in 89.1 [P < 0.001] for HEC1a parental; Fig. 2 C and Fig. S2 D). As the gold-standard functional CSC assay is limiting dilution tumor initiation in vivo, we injected CD55-silenced and nontargeted control CSCs into immune-compromised mice at 10^3^, 10^4^, and 10^5^ cells/mouse (Fig. 2 D). CD55-silenced cells initiated tumors at a frequency of 1 in 78,398 with the first shRNA construct (P < 0.001), and none of the mice injected with the second construct developed tumors (P < 0.001), compared with a frequency of 1 in 4,522 in nontargeted cells (Fig. 2 D). These data provide evidence that CD55 is necessary for CSC maintenance and tumor initiation.

Cisplatin resistance is a hallmark of endometrioid CSCs (Wiechert et al., 2016), and given the high expression of CD55 in CSCs and cisplatin-resistant parental cells, we investigated whether CD55 inhibition affects cisplatin resistance. CD55-silenced CSCs from cisplatin-naive cells lines (A2780, TOV112D) and PDX cells (EEC-4) had significantly higher sensitivity to cisplatin and lower survival rates at cisplatin doses from 2.5 to 50 µM, compared with nontargeted control cells (Fig. 2 E and Fig. S3 A). Further, CD55-silenced CSCs demonstrated higher caspase 3/7 activity compared with nontargeted CSCs upon cisplatin treatment (2.5–10 µM), indicating increased susceptibility to cisplatin-induced cell death (Fig. S3 B). Similarly, CD55 inhibition led to increased sensitivity to cisplatin in cisplatin-resistant CP70 and HEC1a cell lines (Fig. S3, C and D). To further validate the effect of CD55 silencing on cisplatin resistance, we injected CD55-silenced and control CSCs into a total of 45 mice at a concentration of 2 million cells/mouse and waited until each mouse developed a 1-cm tumor (Fig. 2 F). As tumors reached the target size, mice were randomized 2:1 to receive cisplatin (2.5 mg/kg three times a week) and vehicle (DMSO) treatments, respectively. In vehicle control groups, mice with CD55-silenced tumors had significantly lower growth rates compared with nontargeted controls (Fig. 2, F and G; Fig. S3 E). After 17 d of cisplatin treatment, tumors originating from CD55-silenced CSCs were more sensitive to cisplatin as compared with tumors originating from nontargeted CSC controls (Fig. 2 G and Fig. S3 E). Moreover, CD55-silenced tumors demonstrated higher degrees of cell death and tumor regression, inflammatory cell infiltrate, and fibrosis compared with nontargeted controls treated with cisplatin (Fig. 2 H). Although CD59 expression was also increased in endometrioid CSCs and cisplatin-resistant cells, we did not observe any attenuation in CSC marker expression, self-renewal, or enhanced sensitivity to cisplatin upon shRNA silencing of CD59 expression (Fig. S2, E and F; and Fig. S3 F). These findings demonstrate that CD55 is necessary for the maintenance of cisplatin resistance in endometrioid CSCs and cisplatin-resistant cells.

### CD55 is sufficient to drive CSC maintenance and cisplatin resistance

**Figure 3.**
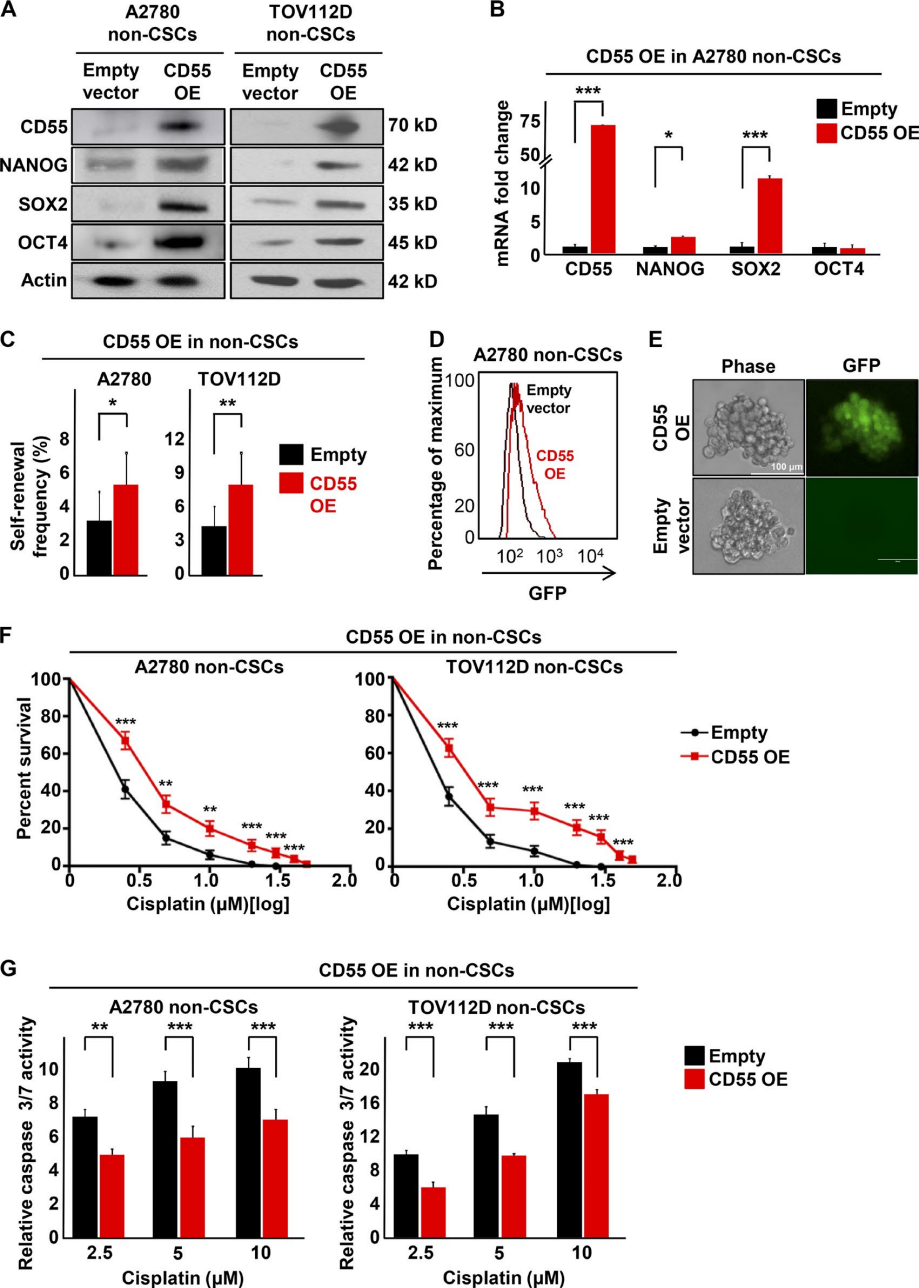
**CD55 is sufficient to drive self-renewal and cisplatin-resistance in endometrioid non-CSCs.** (A) Immunoblots of cisplatin-naive non-CSCs with CD55 overexpression (OE) and empty vector controls were probed with CD55, NANOG, SOX2, and OCT4. Actin was used as loading control. Data are representative of two independent experiments. (B) mRNA expression was determined by quantitative real-time PCR and compared between CD55-overexpressing A2780 non-CSCs and empty vector control non-CSCs. Actin was used as a control. Three technical replicates were used. (C) Limiting dilution analysis plots of empty vector control compared with CD55 overexpression in cisplatin-naive non-CSCs. The graph compares the estimates of the percentage of self-renewal frequency in sorted populations with the corresponding p-values. Data are representative of three independent experiments. (D) A2780 non-CSCs transduced with CD55 overexpression and empty vector controls were flowed for GFP signal intensity, which indicates *NANOG* promoter activity. (E) Tumorsphere from A2780 non-CSCs transduced with CD55 and empty vector control were imaged using a digital immunofluorescence microscope. (F) CD55-overexpressing cisplatin-naive non-CSCs and their empty vector controls were treated with 0–50 µM cisplatin, and percentage of surviving cells was graphed. Data are representative of three independent experiments. (G) Relative caspase 3/7 activity of CD55-overexpressing cisplatin-naive cells and empty vector controls after cisplatin treatment. Relative caspase activities in cisplatin treated groups were calculated after normalizing the corrected readings to untreated controls in each group. Data are representative of two independent experiments, and three technical replicates were used in each. *, P < 0.05; **, P < 0.01; ***, P < 0.001. Bars, 100 µm.

Given the necessary role of CD55 in maintenance of self-renewal and cisplatin resistance, we investigated whether CD55 was sufficient to induce stemness and cisplatin resistance in non-CSCs and cisplatin-naive cells, both of which express low levels of CD55. We successfully transduced CD55 into non-CSCs of cisplatin-naive cells (A2780, TOV112D; Fig. 3 A). Upon CD55 overexpression (CD55 OE), we observed an increase in protein expression of core pluripotency genes (Fig. 3 A). We also observed an increase in NANOG and SOX2 mRNA levels upon CD55 overexpression (Fig. 3 B). Moreover, CD55 overexpression in non-CSCs led to significantly higher self-renewal and stem cell frequencies compared with non-CSCs transduced with empty vector (increased from empty vector to CD55 overexpression conditions as 1 in 33.8 to 1 in 18.8 for A2780 non-CSCs [P < 0.05] and 1 in 23.9 to 1 in 12 for TOV112D non-CSCs [P < 0.01]; Fig. 3 C). Using our NANOG promoter GFP reporter system, which allows direct visualization of stemness, we demonstrated an increase in GFP signal upon CD55 overexpression (Fig. 3 D). Additionally, tumorspheres originating from CD55-overexpressing non-CSCs demonstrated a heterogeneous distribution of GFP signal, compared with empty vector–transduced non-CSCs, which exhibited low GFP signal (Fig. 3 E). We further investigated whether CD55 overexpression was sufficient to induce cisplatin resistance. CD55-overexpressing non-CSCs had significantly higher rates of survival and lower levels of caspase 3/7 activity upon cisplatin treatment compared with non-CSCs transduced with empty vector (Fig. 3, F and G). These data demonstrate that CD55 is sufficient to induce CSC marker expression, self-renewal, and cisplatin resistance in non-CSCs.

### CD55 regulates self-renewal and cisplatin resistance via a complement-independent mechanism

To interrogate the mechanism by which CD55 regulates these phenotypes, we first studied its canonical function in blocking complement cascade. Because our cell culture conditions and NSG mice did not have complement proteins, we assessed this function by conventional BCECF (2′,7′-bis-[2-carboxyethyl]-5-[and-6]-carboxyfluorescein)-based cytotoxicity assay after incubating cells with normal human serum (NHS). We found that non-CSCs and cisplatin-naive (A2780) cells, which had lower levels of CD55, had significantly higher amounts of BCECF leakage compared with their CSC and cisplatin-resistant (CP70) counterparts, respectively (Fig. S4 A). Additionally, CD55+ A2780 cells demonstrated higher proliferative capacity at lower NHS doses compared with CD55− cells (Fig. S4 B). However, complement treatment did not affect self-renewal or cisplatin resistance in CD55+ and CD55− cell populations (Fig. S4, C and D). These data suggested that even though CD55+ cells are more resistant to complement-mediated cytotoxicity, addition of complement does not alter self-renewal or cisplatin resistance, thus indicative of a complement-independent mechanism.

### CD55 function depends on GPI-anchorage to lipid rafts

**Figure 4.**
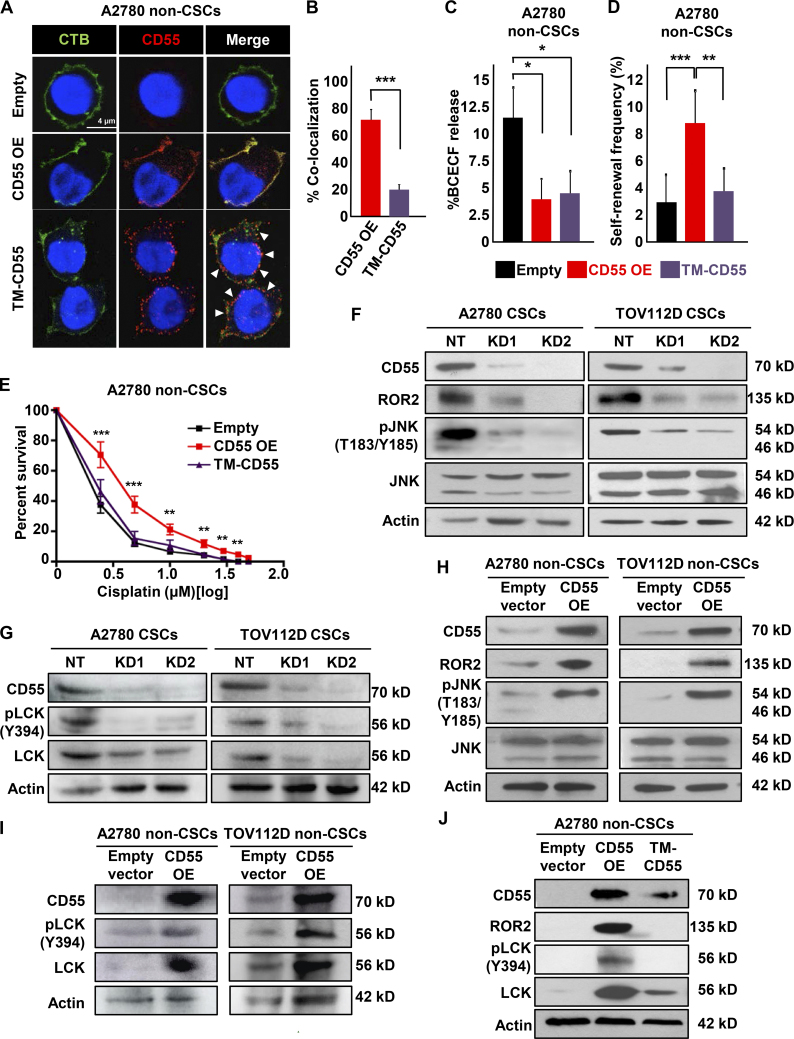
**CD55 localization to lipid rafts is essential for its signaling via ROR2-JNK and LCK pathways.** (A) Immunofluorescent staining of cisplatin-naive non-CSCs transduced with CD55 OE, GPI-deficient transmembrane (TM)-CD55, and empty vector control. The arrowheads point to areas where CD55 is not localized to lipid rafts. (B) Graph showing the percentage of CD55–cholera toxin B colocalization. Data are representative of two independent experiments, quantifying >40 cells/group. (C) Complement-mediated cytotoxicity as assessed by the percentage BCECF dye release in A2780 non-CSCs transduced with CD55 OE, TM-CD55, and empty vector control. Data are representative of two independent experiments, and three technical replicates were used. (D) Limiting dilution analysis plots of CD55 empty vector control compared with CD55 OE and TM-CD55 constructs in cisplatin-naive non-CSCs. (E) CD55 OE cisplatin-naive non-CSCs and their empty vector controls were treated with 0–50 μM cisplatin, and percentage surviving cells was graphed. Data are representative of three independent experiments. (F and G) Immunoblots of cisplatin-naive CSCs silenced for CD55 using two shRNA constructs and a nontargeting control were probed with CD55, ROR2, pJNK (T183/Y185), JNK, pLCK (Y394), and LCK. Actin was used as a loading control. Data are representative of two independent experiments. (H and I) Cell lysates from cisplatin-naive non-CSCs transduced with CD55 and empty vector control were probed for CD55, ROR2, pJNK (T183/Y185), JNK, pLCK (Y394), and LCK. Actin was used as a loading control. Data are representative of two independent experiments. (J) Immunoblots of cisplatin-naive non-CSCs transduced with CD55, TM-CD55, and empty vector control were probed with CD55, ROR2, pLCK (Y394), and LCK. Actin was used as a loading control. *, P < 0.05; **, P < 0.01; ***, P < 0.001. Bar, 4 µm.

It has been reported that GPI-anchored proteins, including CD55, are localized to lipid rafts and can activate nonreceptor tyrosine kinases (Shenoy-Scaria et al., 1992). First, we confirmed that CD55 localized to lipid rafts by co-immunolocalization with cholera toxin B, a marker of lipid rafts (Fig. S4 E). We investigated whether a GPI-deficient transmembrane CD55 (TM-CD55) construct can activate this signaling. We transduced non-CSCs with empty vector control, CD55 OE, or TM-CD55 vectors, with the latter being a chimeric protein containing the extracellular portion of CD55 (amino acids 1–304) fused to the transmembrane and cytoplasmic domains of CD46 (amino acids 270–350; Shenoy-Scaria et al., 1992). In non-CSCs transduced with CD55, the protein localized mainly to the lipid rafts, but TM-CD55 construct was distributed more uniformly on the membrane, with significantly lower level of colocalization with the lipid raft marker (67.5% in CD55-transduced non-CSCs vs. 18.7% in TM-CD55–transduced non-CSCs, P < 0.001; Fig. 4, A and B). Despite the decreased lipid raft localization, non-CSCs transduced with TM-CD55 were resistant to complement-mediated cytotoxicity to the level of CD55-overexpressing non-CSCs (Fig. 4 C). However, TM-CD55 transduced into A2780 non-CSCs demonstrated lower self-renewal and stem cell frequencies compared with CD55 OE (1 in 29.2 in empty vector transduced, 1 in 11.8 in CD55 transduced [P < 0.001], and 1 in 26.4 in TM-CD55 transduced [P < 0.01] non-CSCs), and cisplatin resistance, compared with non-CSCs with CD55 overexpression (Fig. 4, D and E). Moreover, upon phosphatidylinositol-specific phospholipase C (PIPLC)–mediated cleavage of CD55 from membrane, CSCs became more sensitive to cisplatin (Fig. S4 F). Collectively, these findings indicate that CD55 function depends on its anchorage to lipid rafts.

### CD55 activates ROR2 kinase and lymphocyte-specific protein tyrosine kinase (LCK)

To identify intracellular CD55 signaling pathways, we performed a receptor tyrosine kinase activation study using an antibody array against 71 tyrosine kinases (Fig. S4 G). This screen revealed a decrease in levels of ROR2 and lymphocyte-specific protein tyrosine kinase (LCK) in CD55-silenced A2780 CSCs compared with nontargeted CSC control (Fig. S4 G). These results were further validated in cisplatin-naive (A2780 and TOV112D) CSCs, in which CD55 inhibition led to decreased ROR2 and its downstream signaling via JNK pathway activation (Fig. 4 F). Additionally, CD55-silenced CSCs had lower levels of LCK and autophosphorylated active pLCK (Y394) compared with nontargeted CSC controls (Fig. 4 G). CD55+ cells demonstrated higher activity of ROR2 and LCK pathways compared with their CD55− counterparts (Fig. S4 H). We could also induce the activation of these pathways with CD55 overexpression in non-CSCs (Fig. 4, H and I). Although non-CSCs transduced with CD55 demonstrated active ROR2 and LCK signaling, these pathways were not induced in non-CSCs with TM-CD55 (Fig. 4 J). These data demonstrate that CD55 signals through ROR2 and LCK pathways and that this signaling depends on its localization to lipid rafts in endometrioid tumors.

### LIME (LCK-interacting transmembrane adaptor) mediates intracellular CD55 signaling

**Figure 5.**
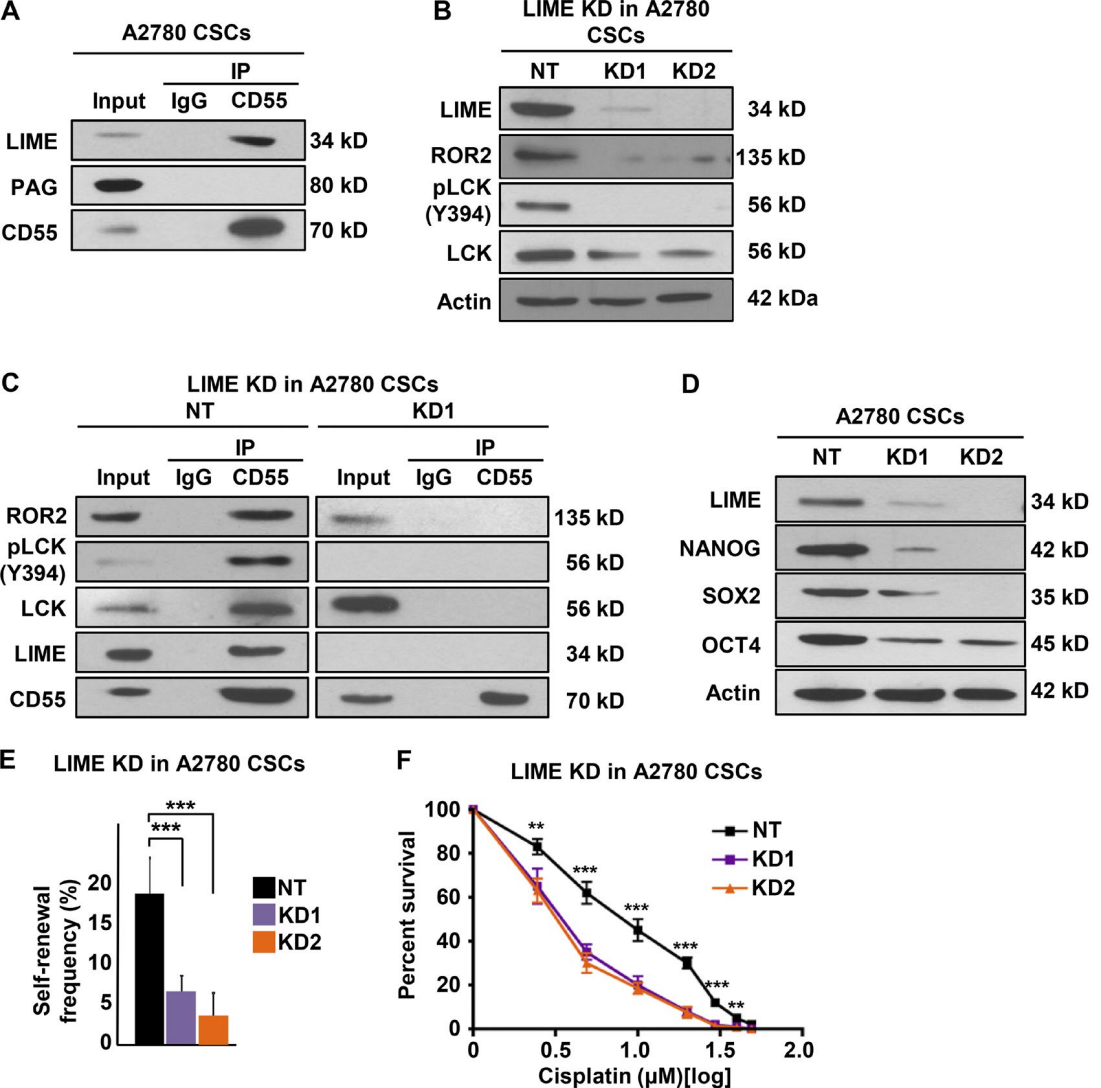
**LIME is necessary for intracellular CD55 signaling.** (A) Immunoprecipitation (IP) experiments with CD55 antibody were performed in cisplatin-naive CSCs, and eluates were probed for lipid raft adaptor proteins LIME and PAG. (B) Cell lysates from LIME-silenced A2780 CSCs and their nontargeted (NT) controls were immunoblotted and probed with LIME, ROR2, pLCK (Y394), and LCK. Actin was used as loading control. (C) IP experiments with CD55 antibody were performed in LIME-silenced and NT control cisplatin-naive CSCs and eluates were probed for ROR2, pLCK (Y394), LCK, LIME, and CD55. (D) Immunoblots of cisplatin-naive CSCs with LIME-silenced and NT controls were immunoblotted for LIME, NANOG, SOX2, and OCT4. Actin was used as a loading control. (E) Limiting dilution analysis of LIME NT control compared with LIME sh1 and sh2 silencing constructs in cisplatin-naive CSCs. (F) LIME-silenced cisplatin-naive CSCs and their NT controls were treated with 0–50 μM cisplatin, and percentage of surviving cells is graphed. All data are representative of two or three independent experiments. **, P < 0.01; ***, P < 0.001.

Because CD55 is an extrinsic protein tethered to the outer membrane with a GPI anchor, we searched for a transmembrane adaptor linking CD55 to signaling molecules located on the inner side of the membrane. We focused on known lipid raft adaptor proteins that were shown to interact with LCK. LIME and PAG (protein associated with glycosphingolipid-enriched microdomains) emerged as candidates (Hořejší et al., 2004; Ventimiglia and Alonso, 2013). To identify CD55-interacting proteins, we performed an immunoprecipitation on endometrioid CSC lysates using CD55 antibodies and immunoblotted for LIME and PAG. We detected LIME but not PAG in the immunoprecipitated lysates (Fig. 5 A). To investigate the functional role of LIME in CD55 signaling, we silenced LIME in CSCs and found decreases in the levels of ROR2, pLCK (Y394), and LCK (Fig. 5 B). Moreover, in LIME-silenced CSCs, CD55 was no longer able to interact with ROR2 and LCK to propagate the signaling (Fig. 5 C). We further assessed the impact of LIME inhibition on self-renewal and cisplatin resistance in CSCs. LIME-silenced cells had lower levels of CSC markers, self-renewal, and stem cell frequencies (1 in 5.2 to 1 in 17.6 and 1 in 22.9, P < 0.001) and higher sensitivity to cisplatin compared with nontargeted control CSCs (Fig. 5, D–F). These data demonstrate that the transmembrane adaptor protein LIME is necessary for intracellular CD55 signaling and maintenance of self-renewal and cisplatin resistance.

### CD55 activates ROR2-JNK signaling to maintain self-renewal

**Figure 6.**
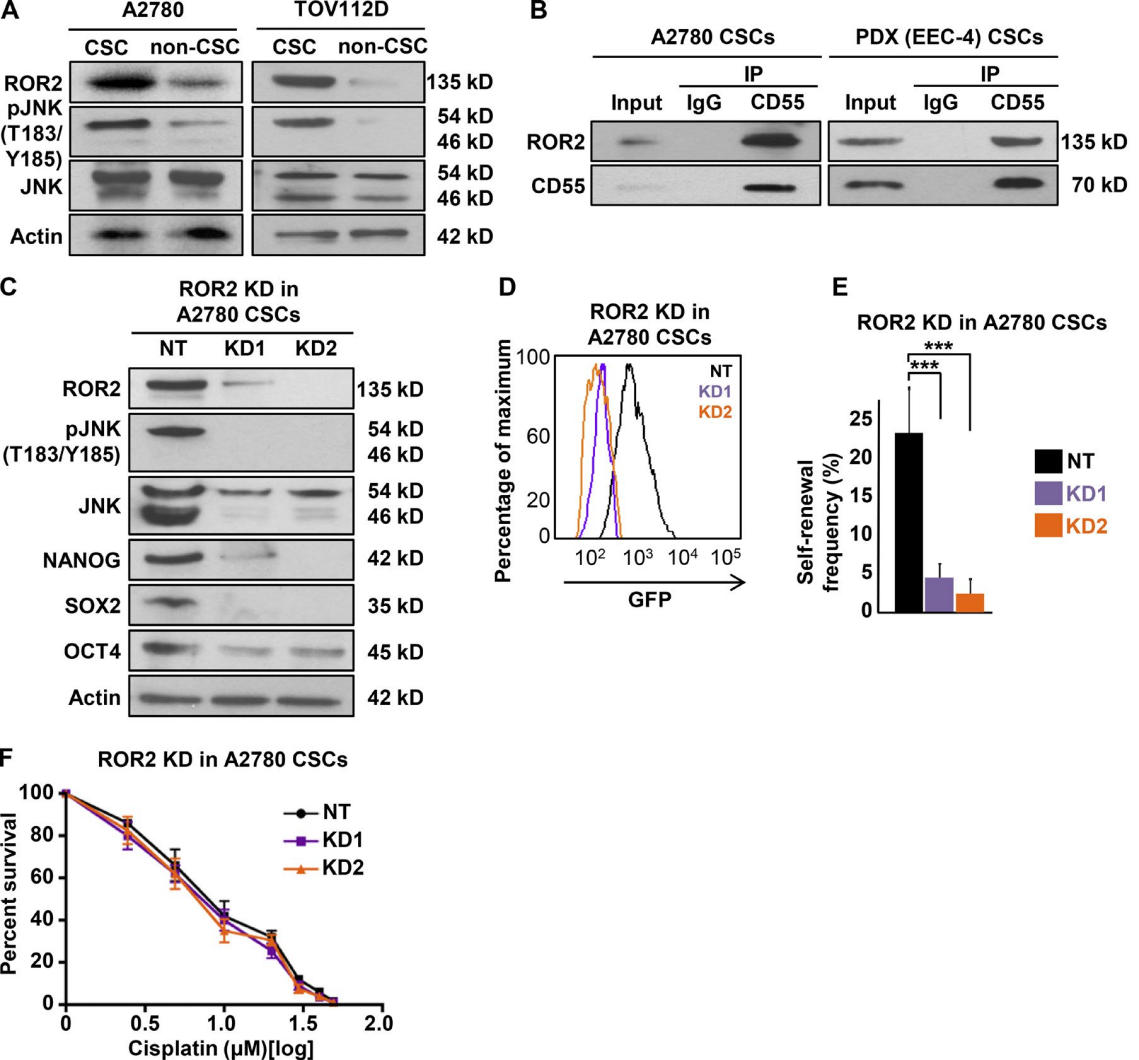
**CD55 signals via ROR2-JNK pathway to regulate self-renewal.** (A) Cell lysates from cisplatin-naive CSCs and non-CSCs were immunoblotted for ROR2, pJNK (T183/Y185), and JNK. Actin was used as a loading control. (B) Immunoprecipitation (IP) analysis with CD55 antibody were performed in cisplatin-naive CSCs, and eluates were immunoblotted for ROR2. (C) Immunoblots of ROR2 silenced using two shRNA constructs and nontargeting constructs in cisplatin-naive CSCs for ROR2, pJNK (T183/Y185), JNK, NANOG, SOX2, and OCT4. Actin was used as a loading control. (D) ROR2 silenced and NT controlled A2780 CSCs analyzed by flow cytometry for GFP intensity, which indicates *NANOG* promoter activity. (E) Limiting dilution analysis of CD55 NT control compared with ROR2 sh1 and sh2 silencing constructs in cisplatin-naive CSCs. (F) ROR2-silenced cisplatin-naive CSCs and their NT controls were treated with 0–50 µM cisplatin, and percentage surviving cells is graphed. All data are representative of two or three independent experiments. ***, P < 0.001.

To elucidate the function of downstream CD55 signaling molecules, we first compared the expression of ROR2 between CSCs and non-CSCs (Fig. 6 A). CSCs of cisplatin-naive cells (A2780 and TOV112D) had higher levels of ROR2 compared with non-CSCs (Fig. 6 A). Both CSCs and non-CSCs demonstrated expression of p46 and p54 JNK isoforms, and the former was higher in CSCs. These isoforms were reported to be protein kinases with no functional difference (i.e., generated with differential mRNA processing) and suggested to be involved in ROR2 signaling (Oishi et al., 2003). In endometrioid tumor cells, only p54 JNK was phosphorylated, and phospho-p54 JNK was higher in CSCs compared with non-CSCs (Fig. 6 A). Given our observation that CD55 silencing led to decreased ROR2 levels and JNK pathway activity in CSCs, whereas CD55 overexpression induced ROR2-JNK signaling pathway, we assessed whether there was a direct or indirect link between CD55 and ROR2. We immunoprecipitated CD55 in A2780 and PDX (EEC-4) CSCs and determined by immunoblotting that ROR2 was coprecipitated (Fig. 6 B). To investigate ROR2 signaling independently, we silenced ROR2 in CSCs, which in turn led to inhibition of p54 JNK phosphorylation and decreases in the levels of core pluripotency transcription factors (NANOG, SOX2, OCT4; Fig. 6 C). We also showed a decrease in GFP intensity of CSCs, which indicated decreased NANOG promoter activity (Fig. 6 D). ROR2-silenced CSCs had significantly lower self-renewal and stem cell frequencies compared with nontargeted CSC controls (decreased from 1 in 4.4 to 1 in 20.7 and 1 in 31.7, P < 0.001; Fig. 6 E). However, ROR2 inhibition did not impact cisplatin resistance in CSCs (Fig. 6 F). Collectively, these data indicate that CD55 interacts with transmembrane ROR2 protein and activates JNK pathway to maintain self-renewal.

### CD55 induces LCK signaling to drive cisplatin resistance

**Figure 7.**
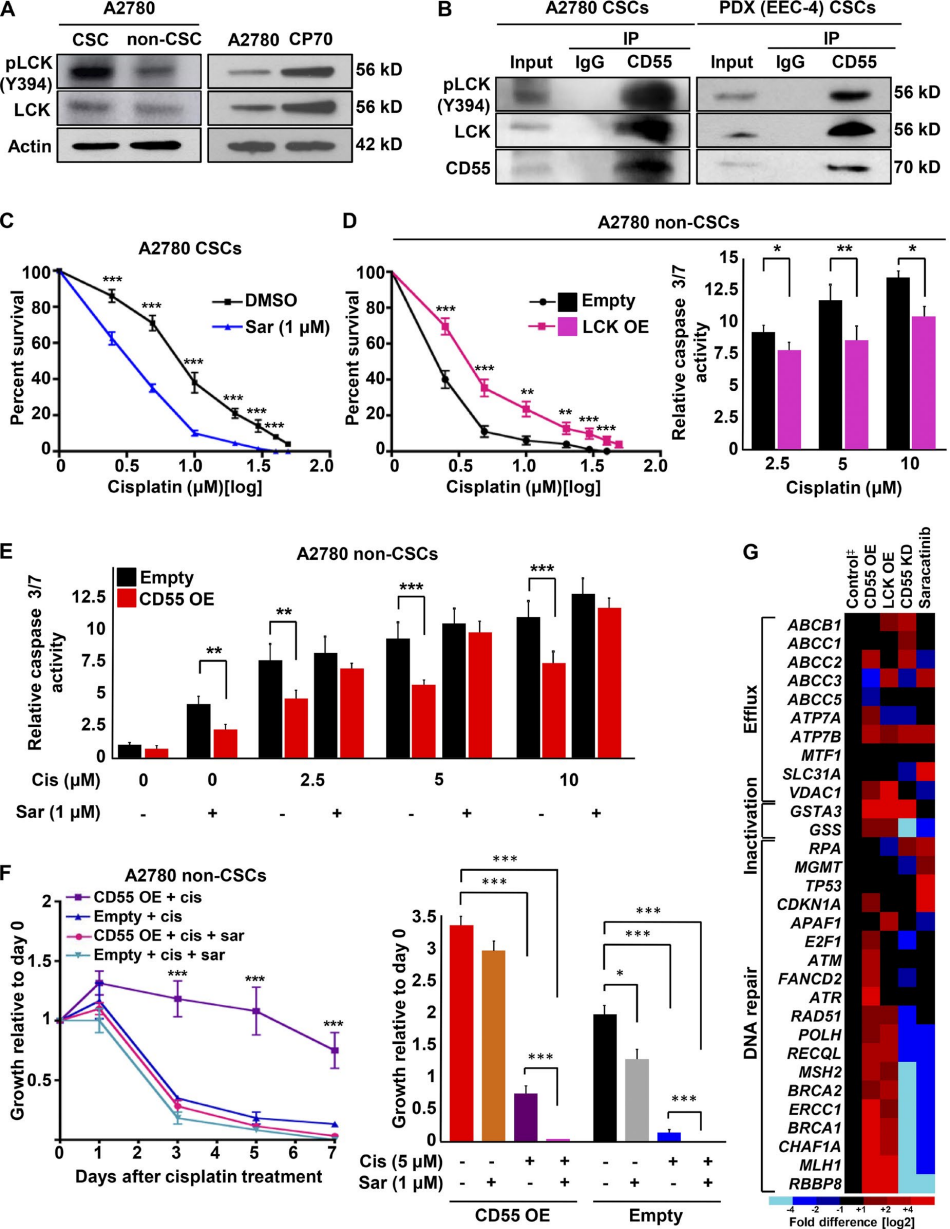
**CD55 signals via LCK pathway to drive cisplatin resistance.** (A) Cell lysates from cisplatin-naive CSCs and non-CSCs were immunoblotted and probed for pLCK (Y394) and LCK. Actin was used as a loading control. (B) Immunoprecipitation (IP) experiments with CD55 antibody were performed in cisplatin-naive CSCs and eluates were probed for pLCK (Y394) and LCK. (C) CSCs were treated with saracatinib (1 µM) or DMSO and then incubated with 0–50 µM cisplatin, and percentage of surviving cells was analyzed. (D) LCK-overexpressing cisplatin-naive non-CSCs and their empty vector controls were treated with cisplatin, and percentage surviving cells and relative caspase 3/7 activity were graphed. OE, overexpression. (E) Relative caspase 3/7 activity for CD55-overexpressing non-CSCs and their empty vector controls treated with or without cisplatin (2.5–10 µM) and with or without saracatinib (1 µM). (F) Growth curves for CD55-overexpressing non-CSCs and their empty vector controls treated with cisplatin with or without saracatinib. The graph shows growth relative to day 0. All data are representative of two or three independent experiments. (G) Targeted gene expression profiling of 31 genes involved in various mechanisms of cisplatin resistance was performed in cisplatin-naive non-CSCs with CD55 or LCK overexpression, and CSCs with CD55 silenced or LCK inhibited with saracatinib. ^‡^Empty vector control for non-CSCs and nontargeted control for CSCs. All data are representative of two independent experiments with three technical replicates. *, P < 0.05; **, P < 0.01; ***, P < 0.001.

Given the finding that CD55 signaling through ROR2-JNK pathway regulates self-renewal alone, we explored the role of LCK, which was the other kinase down-regulated or induced with CD55 silencing and overexpression, respectively. CSCs and cisplatin-resistant cells had higher levels of both pLCK (Y394) and LCK compared with their non-CSC and cisplatin-naive counterparts, respectively (Fig. 7 A and Fig. S5 A). We did not detect phosphorylation of LCK at Y505 residue, which leads to inhibition of LCK, in any of the cells (not depicted). Moreover, when CD55 was immunoprecipitated in A2780 and PDX (EEC-4) CSCs, as well as cisplatin-resistant (CP70) cells, LCK and pLCK (Y394) were coprecipitated (Fig. 7 B and Fig. S5 B). To study the effects of LCK inhibition, we treated CSCs with a FYN/LCK inhibitor, saracatinib, and assessed self-renewal and cisplatin resistance. At 500 nM and 1 µM concentrations of saracatinib, we did not observe a significant change in self-renewal and stem cell frequencies (1 in 1.4 in DMSO control to 1 in 1.8 with 500 nM and 1 in 2.6 with 1 µM saracatinib, P > 0.05; Fig. S5 C). However, CSCs treated with 1 µM saracatinib demonstrated significantly higher sensitivity to cisplatin compared with CSCs treated with cisplatin and DMSO (Fig. 7 C). In addition to pharmacologic inhibition, we silenced LCK in CSCs with three nonoverlapping shRNA constructs (Fig. S5 D). Similarly, LCK-silenced CSCs demonstrated significantly higher sensitivity to cisplatin (Fig. S5 E). To investigate whether LCK is sufficient to drive these phenotypes, we transduced non-CSCs with LCK overexpression and empty control vectors. Although LCK overexpression did not affect the levels of CSC markers and self-renewal in non-CSCs (stem cell frequencies of 1 in 24.1 in empty vector and 1 in 25.5 in LCK overexpression, P > 0.05; Fig. S5, F and G), LCK overexpressing non-CSCs had significantly higher survival rates and lower caspase 3/7 activity levels compared with non-CSCs with empty vector transduction (Fig. 7 D). To assess whether LCK inhibition can overcome CD55-induced cisplatin resistance, we treated CD55-overexpressing and empty vector–transduced non-CSCs with cisplatin and/or 1 µM saracatinib. Although CD55-transduced non-CSCs were more resistant to cisplatin and had lower levels of caspase 3/7 activity, cotreatment with 1 µM saracatinib could overcome the resistance conferred by CD55 (Fig. 7, E and F; Fig. S5 H). To elucidate the particular mechanism of cisplatin resistance activated by CD55-LCK signaling, we performed a targeted screening of 31 genes involved in mechanisms of platinum resistance, including drug efflux, inactivation, and DNA repair (Fig. 7 G). When non-CSCs transduced with CD55 or LCK and CSCs with CD55 silencing and saracatinib treatment were compared with their respective controls (i.e., empty vector, nontargeted control, and DMSO treatment, respectively), genes involved in DNA repair, including *MLH1* and *BRCA1*, were found to be modulated by these modifications (Fig. 7 G and Fig. S5 I). Moreover, upon inhibition of *MLH1* and *BRCA1*, CSCs showed increased sensitivity to cisplatin (Fig. S5, J and K). These data indicate that CD55 signals through LCK pathway to induce cisplatin resistance via increased expression of DNA repair genes. Further, inhibition of this pathway with saracatinib can sensitize cells to cisplatin.

**Figure 8.**
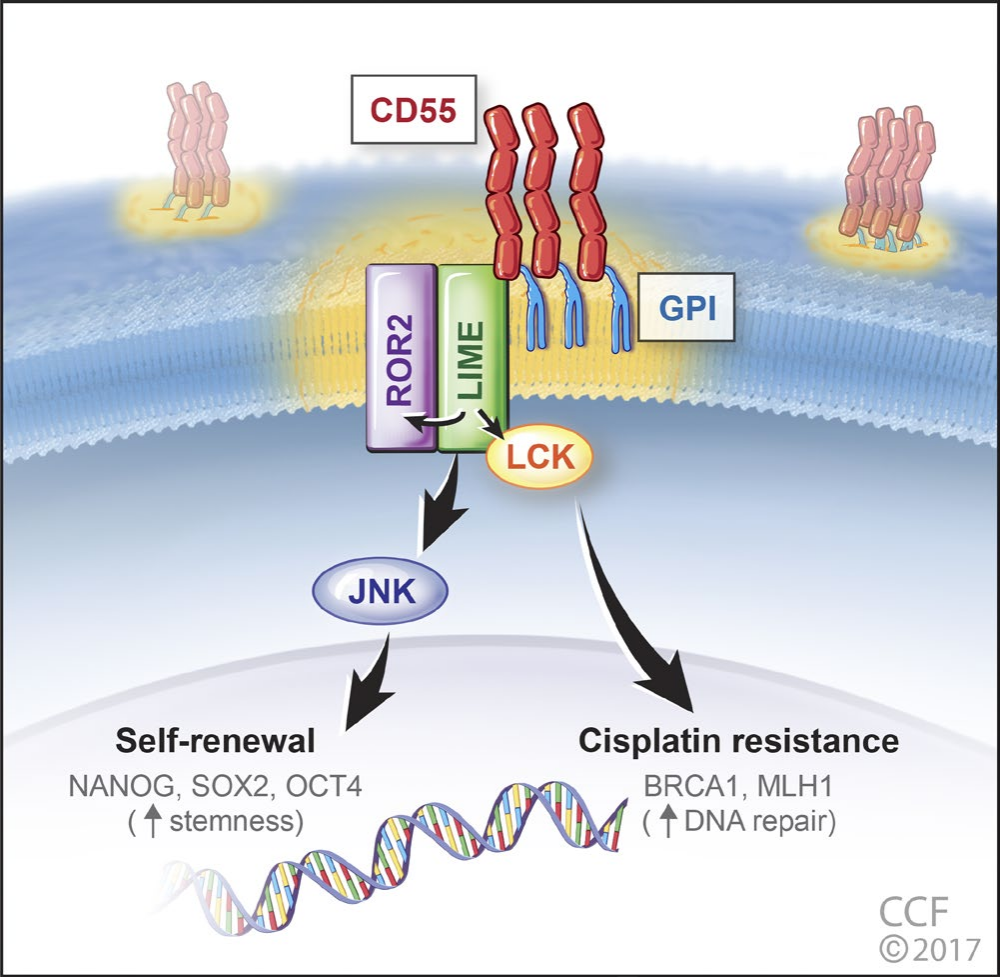
**CD55 regulates self-renewal and cisplatin resistance in endometrioid tumors.** CD55 is glycophosphatidylinositol (GPI)–anchored to lipid rafts and via LIME binding signals intracellularly to ROR2 and LCK. ROR2 via JNK signaling regulates pluripotency gene expression, namely NANOG, SOX2, and OCT4 to maintain stemness in CSCs. In parallel, CD55 via the LCK pathway promotes the expression of DNA repair genes (including BRCA1 and MLH1) to drive cisplatin resistance.

Collectively, these findings demonstrate that CD55 is GPI-anchored to lipid rafts and signals via LIME to activate ROR2-JNK and LCK pathways to regulate self-renewal and cisplatin resistance, respectively (Fig. 8).

## Discussion

These data provide the first evidence of CD55 signaling in a complement-independent manner in solid tumors to regulate self-renewal and therapeutic resistance. Although previous efforts identified CD55 as a prognostic marker in several cancers, our data provide mechanistic insight into a bifurcating signaling network that regulates self-renewal via ROR2/JNK signaling and cisplatin resistance via LCK signaling. Our functional studies demonstrate that CD55 is necessary and sufficient for CSC maintenance. Mechanistically, CD55 regulates the protein expression of the key pluripotency transcription factors NANOG, SOX2, and OCT4, master regulator of CSC self-renewal. CD55 regulates NANOG and SOX2 protein expression at the transcriptional level (Qiu et al., 2010), but regulation of OCT4 expression is more complex and indicative of posttranscriptional regulation, either at the level of protein synthesis or protein stability via phosphorylation, ubiquitination, and/or SUMOylation (Saxe et al., 2009; Yao et al., 2014). Insights into CSC biology have uncovered a series of molecular mechanisms that individually regulate self-renewal and therapeutic resistance, but few signaling networks have the capacity to affect both processes. CD55 represents one such signaling hub that both pathways originate from and hence represents an attractive therapeutic target in endometrioid cancers. In our preclinical studies, we observed that saracatinib sensitized CSC to cisplatin and overcame CD55-induced chemoresistance but did not alter self-renewal. These studies suggest that the downstream CD55 signaling can be targeted with currently available agents but highlights the need for the development of CD55 inhibitors that attenuate both self-renewal and therapeutic resistance. Although the mechanisms that govern self-renewal and therapeutic resistance have traditionally posed barriers to effective treatment with conventional chemotherapy, CD55 intracellular signaling represents a central target that offers an opportunity to prevent recurrence and associated morbidity and mortality in patients with endometrioid cancer.

## Materials and methods

### Cell culture

The isogenic endometrioid ovarian cancer cell lines A2780 (cisplatin-naive) and CP70 (cisplatin-resistant) were cultured in log-growth phase in DMEM supplemented with 10% heat-inactivated FBS (HI-FBS) at 37°C in a humidified atmosphere (5% CO_2_). Endometrioid TOV112D ovarian cancer cell line was cultured in a 1:1 mixture of MCDB 105 medium and Medium 199, supplemented with 15% HI-FBS. Patient-derived primary endometrioid endometrial cancer xenograft (PDX) EEC-4 was a gift from J.J.Kim’s laboratory and maintained in RPMI 1640 with 10% HI-FBS (Unno et al., 2014). Cisplatin-resistant primary endometrial cancer cell line HEC1a was cultured in modified McCoy’s 5a medium. Cell lines were obtained from American Type Culture Collection and authenticated by short tandem repeat DNA profiling analysis. At 70%–90% confluence, trypsin (0.25%)/EDTA solution was used to detach cells for passaging and further experiments until passage number 15. Primary uterine endometrioid cancer cells, UTE-1 and UTE-2, were freshly dissociated from surgical specimens. Cisplatin was obtained from the Cleveland Clinic Hospital pharmacy, and 1 mg/ml stock solutions were stored at 4°C. Saracatinib (AZD0530) was obtained from Selleck Chemicals, and 50 μM stock solutions were stored at −20°C.

### Flow cytometry and high-throughput flow screen

Endometrioid tumor cells at a concentration of 1 million cells/ml were sorted on BD FACS Aria II to isolate CSCs and non-CSCs. For NANOG-GFP sorting, GFP-high and GFP-low populations were sorted from NANOG-GFP promoter transduced stable A2780/CP70 cells as previously described (Wiechert et al., 2016). The antibodies used for FACS analysis were APC-conjugated integrin α6 (1:100; BD Biosciences) and APC-conjugated CD55 (1:100; BD Biosciences). Appropriate isotype controls were used to set gates. Data analysis was performed using FlowJo software (Tree Star).

For high-throughput flow cytometry screening, we used the BD Lyoplate Human Cell Surface Marker Screening Panel, which was purchased from BD Biosciences. The panel contains 242 purified mAbs to cell surface markers and both mouse and rat isotype controls for assessing background signals. For the screening procedure, A2780 and CP70 NANOG-GFP cells were prepared in single-cell suspensions in BD PharMingen Stain Buffer with the addition of 5 mM EDTA. The screening was performed as previously described (Thiagarajan et al., 2015). A2780 and CP70 NANOG-GFP cells were stained with DRAQ5 (eBioscience) and Pacific Blue dyes (Life Technologies), respectively. The cells were then pooled and plated in 96-well plates (BD Biosciences). Reconstituted antibodies were added to the wells as per the human lyoplate screening panel. After the washes, cells were stained with APC-labeled goat anti-mouse IgG secondary antibody (BD Biosciences) and stained using the LIVE/DEAD Fixable Blue Dead Cell Stain kit (Life Technologies). Cells were analyzed using a Fortessa HTS system (BD Biosciences). Data were analyzed using FlowJo software, and appropriate isotype controls were used to detect positive immunoreactivity.

### Immunoblotting and immunoprecipitation

For immunoblots, whole-cell protein extracts were obtained by lysis of cells in 20 mM Tris-HCl, pH 7.5, 150 mM NaCl, 1 mM Na_2_EDTA, 1% NP-40, 1 mM EGTA, 1% sodium pyrophosphate, 1 mM β-glycerophosphate, 1 mM sodium orthovanadate, 1 μg/ml leupeptin, 20 mM NaF, and 1 mM PMSF. Protein concentrations were measured with Bradford reagent (Bio-Rad). Proteins in lysates (30–50 μg total protein) were resolved by 10% SDS-PAGE and transferred to nitrocellulose membrane. Membranes were incubated overnight at 4°C with primary antibodies against CD55 (1:1,000; Santa Cruz), CD59 (1:1,000; Abcam), CD46 (1:1,000; Santa Cruz), NANOG (1:500; Cell Signaling), SOX2 (1:500; Cell Signaling), OCT4 (1:500; Cell Signaling), ROR2 (1:1,000; BD Biosciences), pJNK (1:1,000; T183/Y185; Cell Signaling), JNK (1:1,000; Cell Signaling), pLCK (Y394; 1:1,000; BD Biosciences), LCK (1:1,000; Santa Cruz), LIME (1:1,000; Invitrogen), PAG (1:1,000; Genetex), and β-actin (1:1,000; Cell Signaling). Secondary anti–mouse or anti–rabbit IgG antibodies conjugated to HRP (1:2,000; Thermo Fisher Scientific) were used, and immunoreactive bands were visualized using the ECL Plus from Pierce.

For immunoprecipitation, cells were lysed in 0.5% Triton X-100, 50 mM Tris, pH 7.6, 300 mM NaCl, 1 mM sodium orthovanadate, 5 mM EDTA, 10 μg/ml leupeptin, 10 μg/ml aprotinin, 10 mM iodoacetamide, and 25 μg/ml p-nitrophenyl guanidinobenzoate, as previously described (Shenoy-Scaria et al., 1992). The lysates were spun at 12,000 *g* for 15 min at 4°C. Supernatants were incubated with rabbit antihuman CD55 primary antibody (Santa Cruz) and the corresponding antibody control for 1 h at 4°C. Protein A/G agarose beads (Santa Cruz) were added to lysates, which were subsequently incubated on a rotating mixer overnight at 4°C. The beads were then washed three or four times at 4°C, and Laemmli sample buffer was added to the beads and boiled for 5 min. Immunoblotting was performed using the indicated primary antibodies described above.

### Quantitative real-time PCR

Total RNA was extracted from CSCs and non-CSCs, CD55 knockdown and overexpressing cells and their respective controls, saracatinib-treated cells, and LCK-overexpressing cells using the RNeasy kit (QIAGEN). For mRNA analysis, cDNA was synthesized from 1 μg total RNA using the Superscript III kit (Invitrogen). SYBR Green-based real-time PCR was subsequently performed in triplicate using SYBR-Green master mix (SA Biosciences) on an Applied Biosystems StepOnePlus real-time PCR machine (Thermo Fisher Scientific). For analysis, the threshold cycle (Ct) values for each gene were normalized to expression levels of β-actin. The primers used are listed in Table 1.

**Table 1.** Primers used for quantitative real-time PCR

Name	Direction	Sequence (5′-3′)
β-actin	Forward	AGAAAATCTGGCACCACACC
	Reverse	AGAGGCGTACAGGGATAGCA
CD55	Forward	TCAAGCAACACGGAGTACAC
	Reverse	CCAAGCAAACCTGTCAACG
CD59	Forward	CAGCCGTCAATTGTTCATCTG
	Reverse	AGTACGTTAGCTCATTTTCCCTC
CD46	Forward	CTTGACAGTTTGGATGTTTGGG
	Reverse	TTTTACTTCTCTGTGGGTCTCATC
NANOG	Forward	CCCAAAGGCAAACAACCCACTTCT
	Reverse	AGCTGGGTGGAAGAGAACACAGTT
SOX2	Forward	CACATGAAGGAGCACCCGGATTAT
	Reverse	GTTCATGTGCGCGTAACTGTCCAT
OCT4	Forward	TGAGTCAGTGAACAGGGAATG
	Reverse	AATCTCCCCTTTCCATTCGG
LCK	Forward	GCCATTATCCCATAGTCCCAC
	Reverse	TGTGCAGAGCGATAACCAG

### Limiting dilution assays

For tumorsphere formation assays, the BD FACS Aria II sorter was used to sort cells in duplicate rows of serial dilutions into 96-well ultra-low-attachment plates (Corning) with 200 μL serum-free DMEM/F12 medium per well supplemented with 10 ng/ml epidermal growth factor (Biosource), 20 ng/ml basic fibroblast growth factor (Invitrogen), 2% B27 (Invitrogen), 10 μg/ml insulin, and 1 μg/ml hydrochloride (Sigma-Aldrich). Tumorspheres were counted within 2 wk under a phase contrasted microscope, and data were analyzed using the Extreme Limited Dilution Analysis platform to determine stem cell frequency (http://bioinf.wehi.edu.au/software/elda/; Hu and Smyth, 2009).

### Lentivirus production and infection

Lentiviral shRNAs for CD55 and LCK transductions were prepared as we previously reported (Lathia et al., 2010, 2014). HEK 293T/17 cells were cotransfected with the packaging vectors pMD2.G and psPAX2 (Addgene), and lentiviral vectors directing expression of shRNA specific to *CD55* (TRCN0000057167, TRCN0000255377), *CD59* (TRCN0000057108, TRCN0000057112), *ROR2* (TRCN0000001490, TRCN0000001491), *LIME* (TRCN0000257009, TRCN0000257011), LCK (TRCN0000001598, TRCN0000001599, TRCN0000001600), *MLH1* (TRCN0000040053, TRCN0000040056), *BRCA1* (TRCN0000039834, TRCN0000039835), a nontargeting (NT) control shRNA (SHC002), and overexpression vector for *CD55*, *LCK*, or an empty vector (Applied Biological Materials). Media of the HEK 293T/17 cells were changed 18 h after transfection, and viral particles were harvested at 48 h via concentration with polyethylene glycol precipitation and stored at −80°C for future use. Viral infections were performed in endometrioid tumor cell lines and PDX cells, and after transduction, cells were selected using 2–5 μg/ml puromycin.

### Cell survival and caspase 3/7 activity assays

Endometrioid CSCs, non-CSCs, and cisplatin-resistant cells were plated in 12-well plates at 50,000 cells/well density and treated on the next day with cisplatin at the doses of 0–50 μM and/or 1 μM saracatinib. The number of live cells in control and treatment groups were manually counted using a hemocytometer at days 5 and 7 using trypan blue dye exclusion as a live cell marker. Percentages of surviving cells at different treatment doses were normalized to the untreated control.

Apoptosis was measured using the Caspase-Glo 3/7 Assay kit (Promega) according to the manufacturer’s instructions. Measured caspase activities were corrected for viable cell density as assessed by CellTiter-Glo (Promega). Relative caspase activities in cisplatin treated groups were calculated after normalizing the corrected readings to untreated controls in each group.

### Xenograft studies

NSG (NOD severe combined immunodeficient (SCID) IL2R gamma) mice were purchased from the Biological Resources Unit at the Lerner Research Institute of the Cleveland Clinic and maintained in microisolator units with free access to water and food. For in vivo tumor initiation assay, CD55-knockdown and NT control A2780 CSCs were transplanted subcutaneously in serial dilutions of 1,000, 10,000, and 100,000 cells (five mice per group) into the right subcutaneous flank of female mice at 6 wk of age. Mice were monitored every day until the endpoint of day 30, when the tumors that were palpable with a cross-sectional area >2 mm^2^ were taken as a positive read. Mice were euthanized, and the tumors were resected. The stem cell frequencies were calculated using the extreme limited dilution analysis algorithm as described above.

For the cisplatin treatment studies, NSG mice were injected subcutaneously with CD55-knockdown and NT control A2780 CSCs (15 mice per group). Each mouse was transplanted with 2 million cells to ensure tumor formation, and tumors were allowed to grow to 1 cm in largest diameter. Then, mice were randomized into two groups, and one group (10 mice) was treated intraperitoneally with cisplatin (2.5 mg/kg three times per week), while the other group (5 mice) received vehicle (DMSO). Tumor size was assessed at indicated time points by caliper measurements of length and width and the volume was calculated according to the formula (length × width^2^/2). Treatments were continued until day 14 in the vehicle arm and day 17 in the cisplatin arm, at which time the mean tumor size reached 2 cm. Mice were euthanized, and the tumors were resected for staining with hematoxylin and eosin. All mouse procedures were performed under adherence to protocols approved by the Institutional Animal Care and Use Committee at the Lerner Research Institute, Cleveland Clinic.

### Complement-mediated cytotoxicity assay

A2780/CP70 parental cell, CSC, and non-CSC cytotoxicity after incubation with serum was assessed by BCECF leakage assay as previously described (Li and Lin, 2012). First, 2 × 10^5^ cells were labeled by incubation with 5 μM of BCECF-AM (Invitrogen) for 30 min at 37°C. After washing, the labeled endometrioid tumor cells were incubated with 10–30% NHS or respective controls in 100 µl of GVB^2+^ buffer for another 30 min at 37°C. Then, supernatants were collected, and BCECF dye release was measured by a fluorescence microtiter plate reader (Molecular Devices) with excitation and emission wavelengths of 485 and 538 nm, respectively. The percentage of BCECF release (indicative of complement mediated injury) was calculated with the following formula: [(A − B)/(C − B)] × 100%, where A represents the mean experimental BCECF release, B represents the mean spontaneous BCECF release (in the absence of serum), and C represents the mean maximum BCECF released that was induced by incubating cells with 0.5% Triton X.

### Immunocytochemistry

To visualize the expression and localization of CD55 and cholera toxin B, a lipid raft marker, A2780 and TOV112D CSCs were plated on coverslips placed in a 6-well plate. After 12–16 h, the cells were fixed for 15 min with 4% paraformaldehyde at room temperature and washed three times with PBS. After washing, cells were incubated with A488-conjugated cholera toxin B (Invitrogen) for 15 min and washed again three times. Then they were blocked in 5% goat serum with 1 mg/ml BSA for 2 h. Mouse monoclonal CD55 antibody (Santa Cruz) was used to stain cells overnight at 4°C. The following day, cells were washed three times with PBS for 5 min, and A647-conjugated goat anti–mouse secondary antibody (Invitrogen) was applied for 1 h at room temperature. After secondary antibody incubation, cells were washed three times with PBS for 5 min each and counterstained with DAPI for 5 min. Afterward, cells were washed three times with PBS for 5 min each. The coverslips were mounted using 50% glycerol, and cells were imaged using a Leica TCS SP8 Confocal/Multi-Photon high-speed upright microscope.

### Generation of GPI-deficient transmembrane CD55 construct

A GPI-deficient transmembrane CD55 (TM-CD55) construct was generated as described in the literature (Shenoy-Scaria et al., 1992). In brief, TM-CD55 consisted of the extracellular portion of CD55 (amino acids 1–304) fused to the transmembrane and cytoplasmic domains of CD46 (membrane cofactor protein; amino acids 270–350). First, the region of CD55 cDNA from amino acids 1–304 was amplified using the specific primers (forward: 5′-ATGACCGTCGCGCGGCC-3′; reverse: 5′-AACATTTACTGTGGTAGGTTTC-3′). Next, the region of CD46 cDNA from amino acids 270–350 was amplified using the specific primers with a stop codon added in the primer (forward: 5′-TGTGACAGTAACAGTACTTGG-3′; reverse: 5′-TCAAATCACAGCAATGACCC-3′). Then, the two PCR products were mixed in equal proportions and a single fusion/chimeric PCR product was generated using Mega PCR. The generated chimeric cDNA PCR product was cloned into pENTR/Directional TOPO vector and then recombined into pLenti-CMV-Puro-Dest vector (Addgene). For transformation, competent *Escherichia coli* strain DH5α was used to introduce 100 ng plasmid via heat shock at 42°C for 45 s. Bacterial colonies resistant to ampicillin were selectively grown, and lentivirus was produced and cells were infected as described above.

### PIPLC treatment

To release CD55 from the lipid rafts, CSCs were treated with the enzyme PIPLC (Sigma-Aldrich) at a final concentration of 4 U/ml and compared with untreated cells. One unit of PIPLC liberates 1 U of acetylcholinesterase per minute at pH 7.4 at 30°C.

### Receptor tyrosine kinase array

For the receptor tyrosine kinase activation study, a RayBio antibody array against 71 unique tyrosine kinases (Raybio AAH-PRTK-1-4) was used according to the manufacturer’s protocol. Cell lysates (1 mg) from A2780 CSCs transduced with NT and two nonoverlapping CD55 knockdown shRNAs were added to each membrane. Spot quantitation was done using ImageJ, and mean densities were calculated for each spot in a duplicate and normalized to the densities of background and positive control dots.

### Gene expression profiling

To identify genes responsible for CD55-mediated regulation of cisplatin resistance, we performed a targeted screening of 31 genes involved in various mechanisms of platinum resistance including drug influx/efflux, inactivation, and DNA repair (Galluzzi et al., 2014). RNA lysates from A2780 CSCs from CD55 silenced versus NT control, saracatinib versus vehicle treatment, and non-CSCs with CD55 OE versus empty vector control, LCK overexpression versus empty control were used to perform serial RT-PCRs in triplicates, and the relative amount of cDNA was calculated by the comparative CT method using actin sequence as the loading control. Fold differences in gene expression were plotted in a heat map. Primer sequences are listed in Table 2.

**Table 2.** Primers used for gene expression profiling

Name	Sequence (5′-3′)
ABCB1-F	CTTCAGGGTTTCACATTTGGC
ABCB1-R	GGTAGTCAATGCTCCAGTGG
ABCC1-F	ACTTCGTTCTCAGGCACATC
ABCC1-R	TGATCCGAAATAAGCCCAGG
ABCC2-F	TCATCGTCATTCCTCTTGGC
ABCC2-R	ACGGATAACTGGCAAACCTG
ABCC3-F	ACCTGTCCAAGCTCAAGATG
ABCC3-R	GGGTGACAAAGAAAACAGGG
ABCC5-F	CAGAGACCGTGAAGATTCCAAG
ABCC5-R	TGAGCTGAGAATGCATGGAG
ATP7A-F	TTGGAAAAGTGAATGGTGTGC
ATP7A-R	GATAACAGCATCAAAGCCCATG
ATP7B-F	GCTCTTTGTGTTCATTGCCC
ATP7B-R	GAGACATGAGTTTAGCCAGGG
MTF1-F	CTTCCTTACCTCTTACAGCCTC
MTF1-R	TGTGAAGCCTCTGATGTGC
SLC31A1-F	GACGGGTTAAGATTCGGAGAG
SLC31A1-R	AGGTTGCATGGTACTGTTGG
VDAC1-F	CCTTCGATTCATCCTTCTCACC
VDAC1-R	GTAACCTAGCACCAGAGCAC
GSTA3-F	AAGTCGCTATTTCCCTGCC
GSTA3-R	GAAGTTGGAGATAAGGCTGGAG
GSS-F	AGCGTGCCATAGAGAATGAG
GSS-R	ATCCCGGAAGTAAACCACAG
RPA-F	CTATAATGAAGGACTCGGGCAG
RPA-R	GTCTTTGAAGCACCATAAGCC
MGMT-F	GCTGAATGCCTATTTCCACC
MGMT-R	CACTTCTCCGAATTTCACAACC
TP53-F	GCCATCTACAAGCAGTCACAG
TP53-R	TCATCCAAATACTCCACACGC
CDKN1A-F	TGTCACTGTCTTGTACCCTTG
CDKN1A-R	GGCGTTTGGAGTGGTAGAA
APAF1-F	GGCTGTGGGAAGTCTGTATTAG
APAF1-R	CAACCGTGTGCAAAGATTCTG
E2F1-F	TCTCCGAGGACACTGACAG
E2F1-R	ATCACCATAACCATCTGCTCTG
ATM-F	ATTCCGACTTTGTTCCCTCTG
ATM-R	CATCTTGGTCCCCATTCTAGC
FANCD2-F	GGAGTCCATGTCTGCTAAAGAG
FANCD2-R	CAATGTGCTTTAACCGAGTGAG
ATR-F	CCTTGAACATGAAAGCCTTGG
ATR-R	CCTGAGTGATAACAGTAGACAGC
RAD51-F	GTGGTAGCTCAAGTGGATGG
RAD51-R	GGGAGAGTCGTAGATTTTGCAG
POLH-F	CTACTCGGGAACAGGTACAATG
POLH-R	ACACGAATGCTCACAACCAG
RECQL-F	AGTTCAGACCACTTCAGCTTG
RECQL-R	GGGCAAATGACGAGTGTAAAAC
MSH2-F	AAAGGGAGAGCAGATGAATAGTG
MSH2-R	TGATTACCGCAGACAGTGATG
BRCA2-F	TTCATGGAGCAGAACTGGTG
BRCA2-R	AGGAAAAGGTCTAGGGTCAGG
ERCC1-F	AATTTGTGATACCCCTCGACG
ERCC1-R	TGTGAGATGGCATATTCGGC
BRCA1-F	GCCTTCTAACAGCTACCCTTC
BRCA1-R	CTTCTGGATTCTGGCTTATAGGG
CHAF1A-F	GAGGATGAAGATGAGGACGATG
CHAF1A-R	TCCTTGGCCTTCAGTTTCTG
MLH1-F	GGCACAGCATCAAACCAAG
MLH1-R	CAAGCATGGCAAGGTCAAAG
RBBP8-F	GAAATTGGCTTCCTGCTCAAG
RBBP8-R	TTTTGGACGAGGACAAGGATC

### Statistical analysis

Values reported in the results are mean ± SD. One-way ANOVA was used to calculate statistical significance, and p-values are detailed in the text and figure legends.

### Online supplemental material

Fig. S1 includes additional data on high CD55 expression in CSCs of cisplatin-naive and cisplatin-resistant cells, including freshly dissociated primary endometrioid uterine cancer specimens. Figs. S2 and S3 show additional data supporting the role of CD55 in maintaining endometrioid tumor self-renewal and cisplatin resistance. Fig. S4 provides data supporting the complement-independent function of CD55. Fig. S5 shows that CD55 induces DNA repair genes to regulate cisplatin resistance.

## Supplementary Material

Supplemental Materials (PDF)
