# Correction: CD55 regulates self-renewal and cisplatin resistance in endometrioid tumors

**DOI:** 10.1084/jem.2017043806042026c

**Published:** 2026-07-30

**Authors:** Caner Saygin, Andrew Wiechert, Vinay S. Rao, Ravi Alluri, Elizabeth Connor, Praveena S. Thiagarajan, James S. Hale, Yan Li, Anastasia Chumakova, Awad Jarrar, Yvonne Parker, Daniel J. Lindner, Anil Belur Nagaraj, J. Julie Kim, Analisa DiFeo, Fadi W. Abdul-Karim, Chad Michener, Peter G. Rose, Robert DeBernardo, Haider Mahdi, Keith R. McCrae, Feng Lin, Justin D. Lathia, Ofer Reizes

Vol. 214, No. 9 | https://doi.org/10.1084/jem.20170438 | August 24, 2017

The authors regret that, during figure preparation, errors were made in Fig. 1 and Fig. 4. In Fig. 1 D, the TOV112D CD59 blot was inadvertently copied from the wrong film. In Fig. 4 I, the TOV112D non-CSC actin blot was accidentally duplicated from Fig. 7 A. The original and corrected figures are shown here. These corrections do not change the original conclusions of the article, and the figure legends remain unchanged. The HTML and PDF versions of this article have been corrected. The errors remain only in print and in PDFs downloaded before July 28, 2026.

**Figure:**
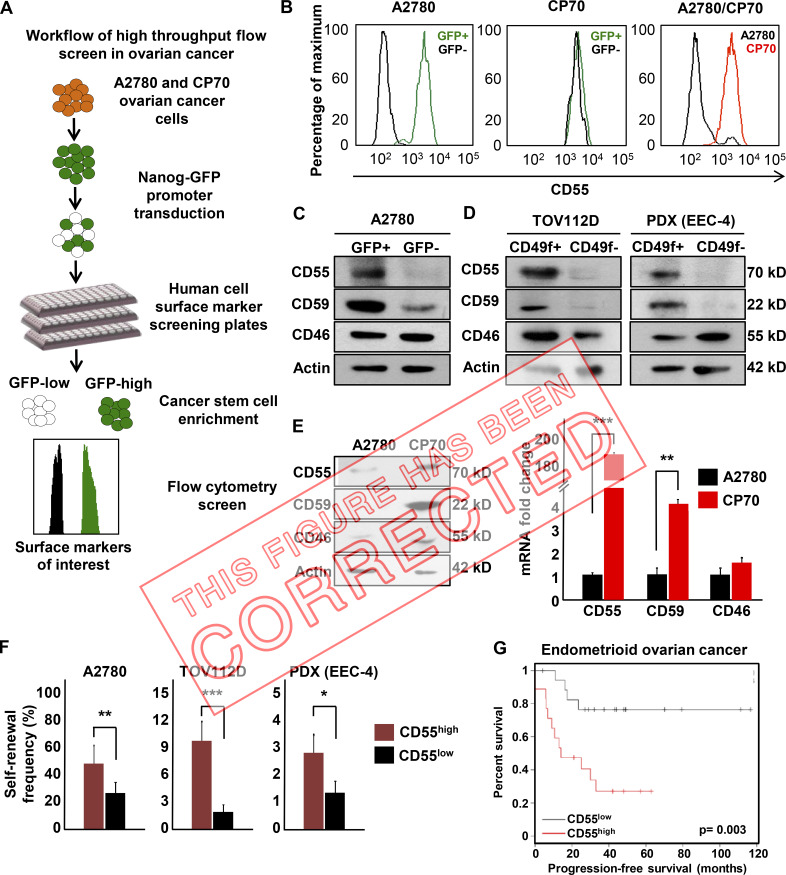


**Figure 1.**
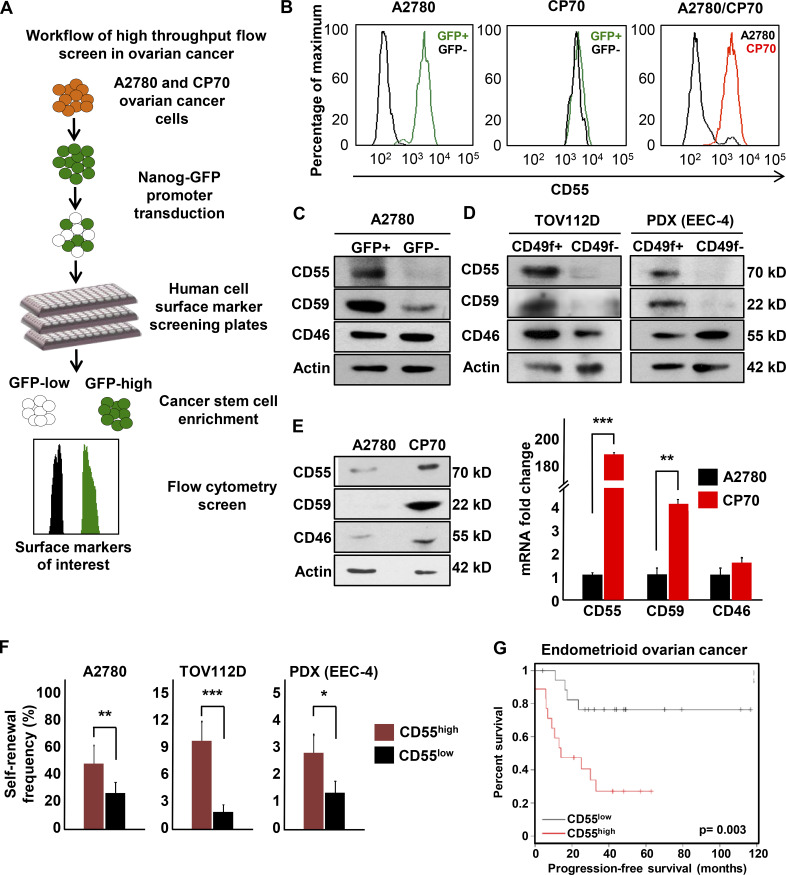
**CD55 is highly expressed on endometrioid ovarian and uterine CSCs and cisplatin-resistant cells. (A)** A high-throughput flow cytometry screen of 242 different surface CD markers in cisplatin-naive (A2780) and cisplatin-resistant (CP70) ovarian cancer cells was performed to investigate the differential expression of these markers between CSCs versus non-CSCs and cisplatin-naive versus cisplatin-resistant cells. **(B)** Of 242 markers, CD55 was the most highly and differentially expressed between cisplatin-naive CSCs versus non-CSCs and cisplatin-resistant versus cisplatin-naive cells. **(C and D)** Cell lysates from cisplatin-naive A2780 reporter, TOV112D, and PDX (EEC-4) cells sorted into CSCs and non-CSCs by GFP expression and CD49f expression, respectively, were probed with anti-CD55, CD59, and CD46 antibodies. Actin was used as a loading control. Data are representative of three independent experiments. **(E)** Protein and mRNA expression of CD55, CD59, and CD46 were assessed in lysates from cisplatin-naive (A2780) and cisplatin-resistant (CP70) cells. Actin was used as a control. Data are representative of two independent experiments. **(F)** Limiting dilution analysis of CD55^+^ compared with CD55^−^ cisplatin-naive cells. The graph represents the estimates in percentage of self-renewal frequency in sorted populations with the corresponding P values. Data represent two independent experiments. **(G)** Kaplan–Meier (K–M) progression-free survival curve for endometrioid ovarian cancer patients who had high versus low tumor CD55 expression before therapy was obtained from K–M plotter database (http://kmplot.com/analysis/). *, P < 0.05; **, P < 0.01; ***, P < 0.001.

**Figure:**
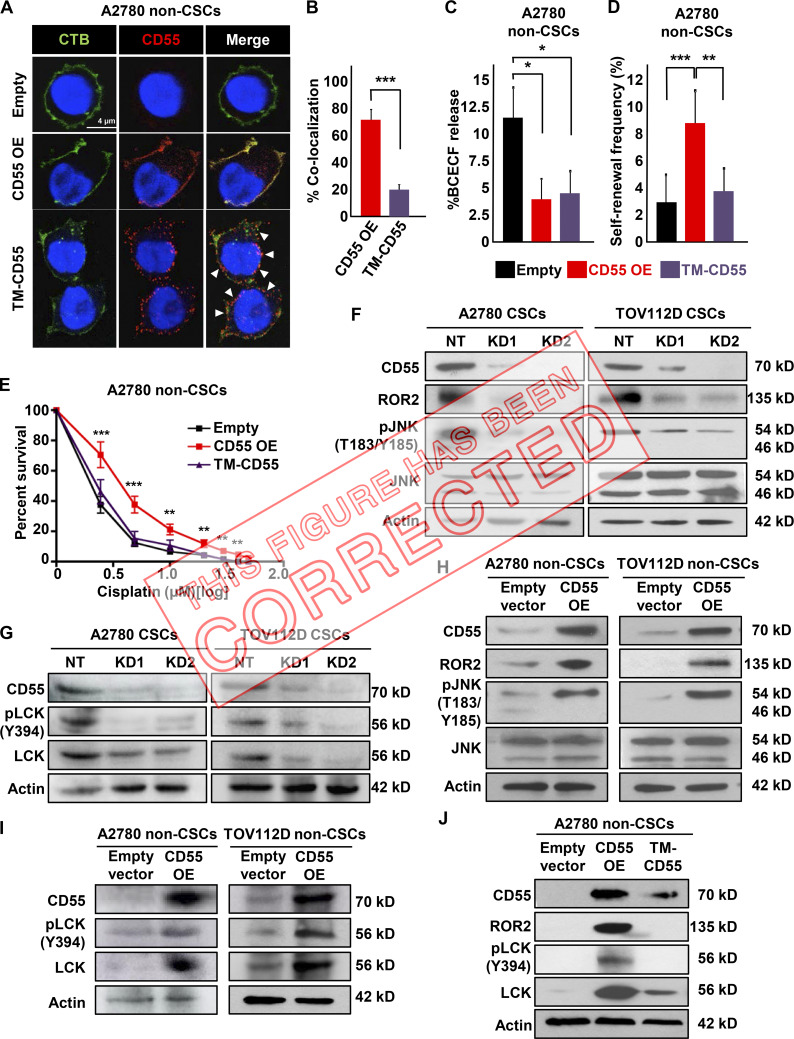


**Figure 4.**
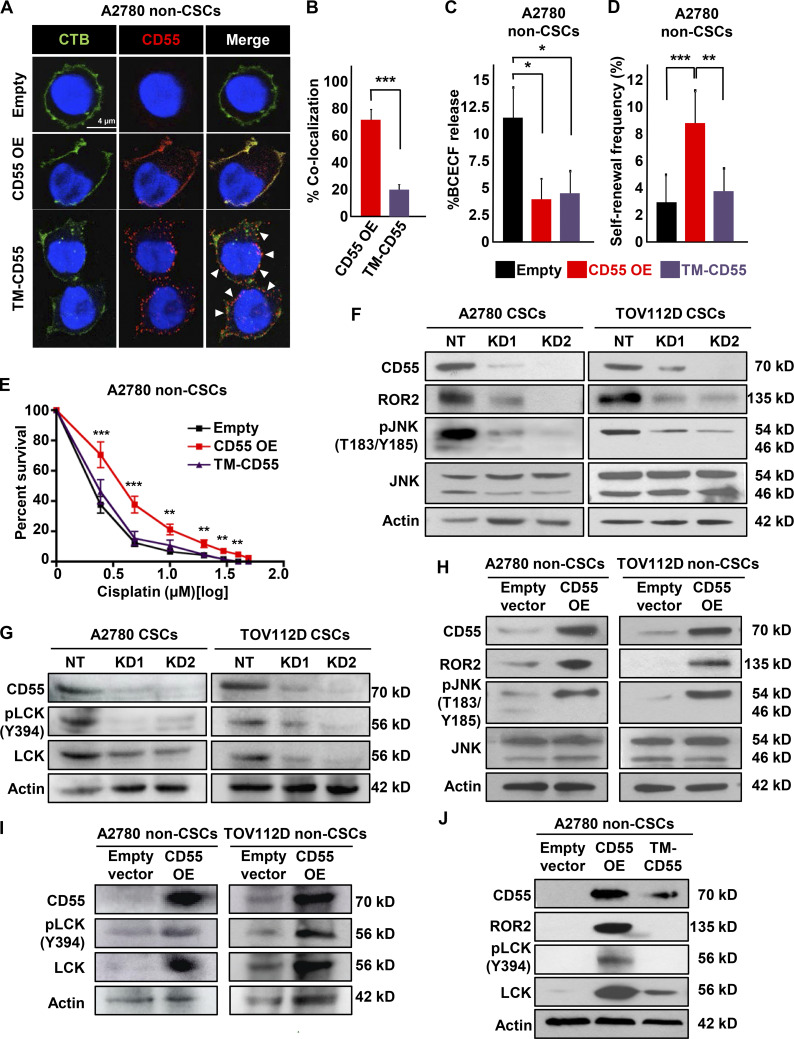
**CD55 localization to lipid rafts is essential for its signaling via ROR2–JNK and LCK pathways. (A)** Immunofluorescent staining of cisplatin-naive non-CSCs transduced with CD55 OE, GPI-deficient transmembrane (TM)-CD55, and empty vector control. The arrowheads point to areas where CD55 is not localized to lipid rafts. **(B)** Graph showing the percentage of CD55–cholera toxin B colocalization. Data are representative of two independent experiments, quantifying >40 cells/group. **(C)** Complement-mediated cytotoxicity as assessed by the percentage BCECF dye release in A2780 non-CSCs transduced with CD55 OE, TM-CD55, and empty vector control. Data are representative of two independent experiments, and three technical replicates were used. **(D)** Limiting dilution analysis plots of CD55 empty vector control compared with CD55 OE and TM-CD55 constructs in cisplatin-naive non-CSCs. **(E)** CD55 OE cisplatin-naive non-CSCs and their empty vector controls were treated with 0–50 μM cisplatin, and the percentage surviving cells was graphed. Data are representative of three independent experiments. **(F and G)** Immunoblots of cisplatin-naive CSCs silenced for CD55 using two shRNA constructs and a nontargeting control were probed with CD55, ROR2, pJNK (T183/Y185), JNK, pLCK (Y394), and LCK. Actin was used as a loading control. Data are representative of two independent experiments. **(H and I)** Cell lysates from cisplatin-naive non-CSCs transduced with CD55 and empty vector control were probed for CD55, ROR2, pJNK (T183/Y185), JNK, pLCK (Y394), and LCK. Actin was used as a loading control. Data are representative of two independent experiments. **(J)** Immunoblots of cisplatin-naive non-CSCs transduced with CD55, TM-CD55, and empty vector control were probed with CD55, ROR2, pLCK (Y394), and LCK. Actin was used as a loading control. *, P < 0.05; **, P < 0.01; ***, P < 0.001. Bar, 4 µm.

