# Supplementary material for: CD55 regulates self-renewal and cisplatin resistance in endometrioid tumors
Source: J Exp Med. 2017 Aug 24;214(9):2715–32. doi: 10.1084/jem.20170438 (PMC5584126; doi:10.1084/jem.20170438)
Supplement: Supplemental Materials (PDF) [file jem_20170438_sm.pdf]

The Journal of Experimental Medicine

SUPPLEMENTAL MATERIAL

Saygin et al., https://doi.org/10.1084/jem.20170438

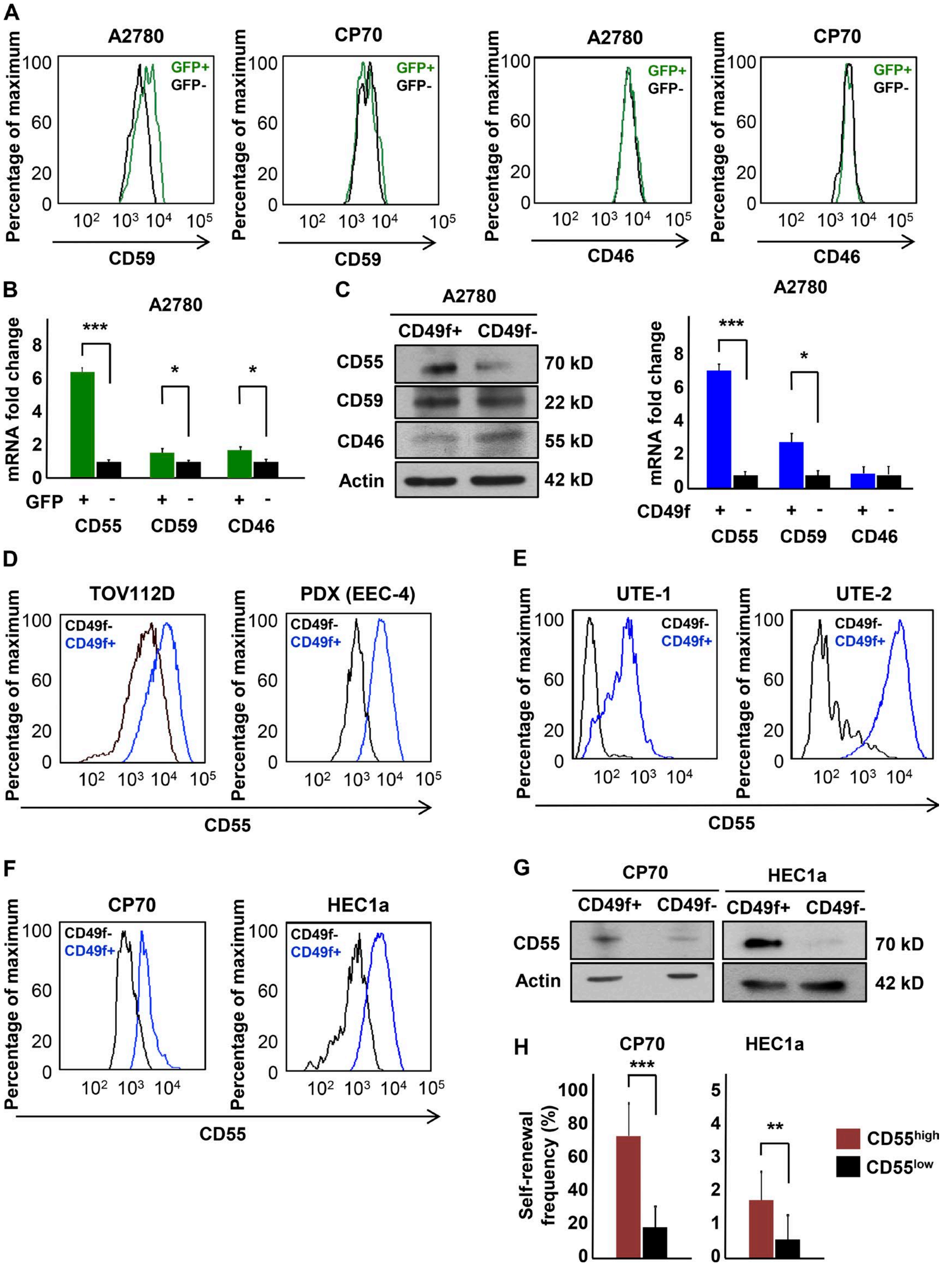

Figure S1. **CD55 is highly expressed on CSCs.** (A) CSC and non-CSC histograms for additional membrane-bound complement inhibitory proteins CD59 and CD46. (B) mRNA expression was determined by quantitative real-time PCR and compared between GFP+ (CSCs) and GFP− (non-CSCs) enriched from A2780 cells using the NANOG-GFP reporter system. Actin was used as a control. (C) CSCs were also enriched by surface CD49f expression in A2780, which demonstrated higher CD55 levels at protein and mRNA levels. (D–F) Cisplatin-naive and cisplatin-resistant CSCs versus non-CSCs histogram plots for CD55 expression. (G) Cell lysates from cisplatin-resistant CSCs and non-CSCs were immunoblotted for CD55. Actin was used as a loading control. (H) Limiting dilution analysis plots of CD55+ and CD55− cisplatin-resistant cells. The graph compares the estimates of the percentage of self-renewal frequency in these sorted populations with the corresponding p-values. *, $P < 0.05$; **, $P < 0.01$; ***, $P < 0.001$.

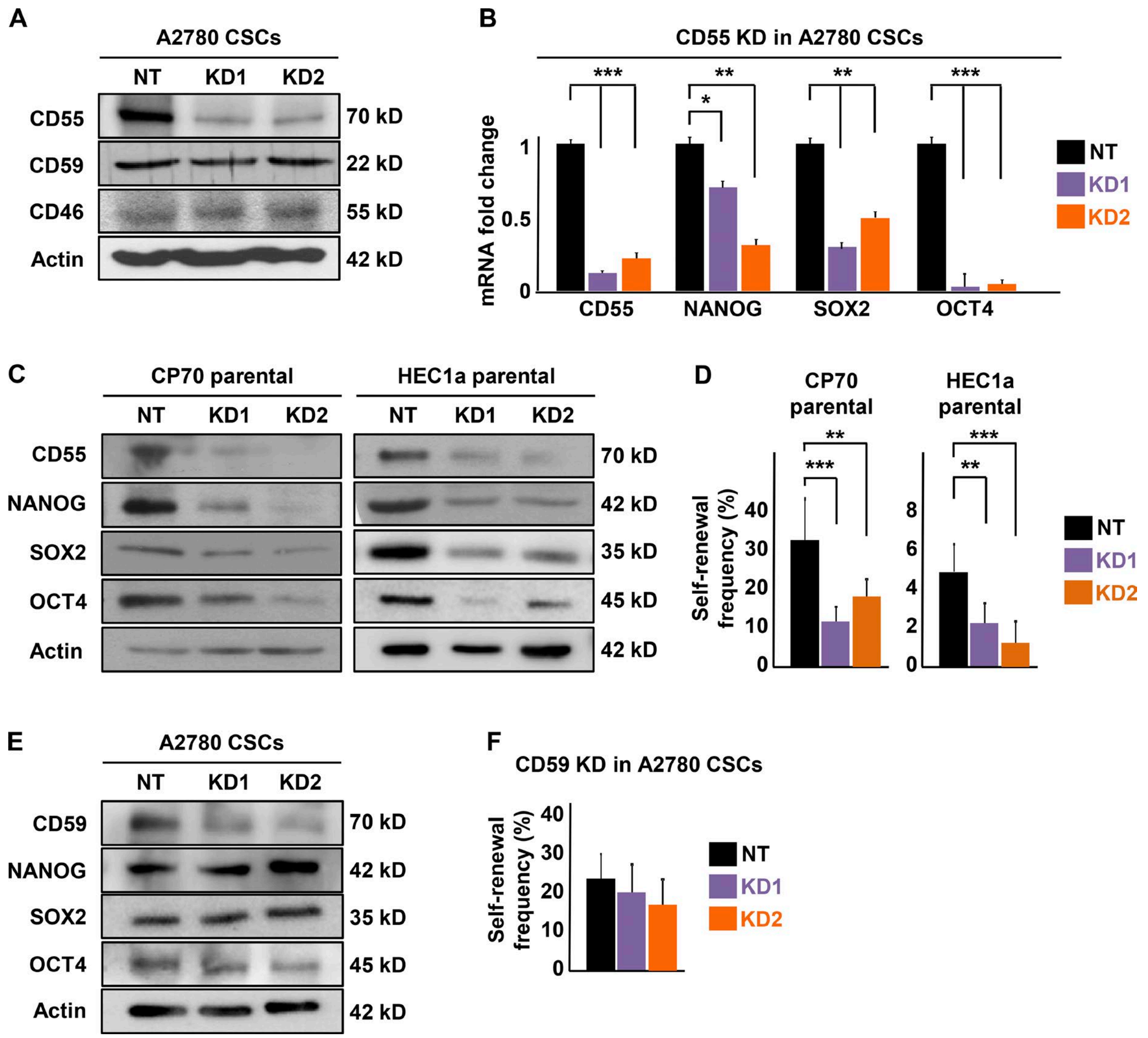

Figure S2. **CD55 maintains self-renewal in cisplatin-resistant endometrioid tumors.** (A) Immunoblots of cisplatin-naive A2780 CSCs with CD55 silencing and nontargeted (NT) control for CD55, CD59, and CD46. Actin was used as loading control. (B) mRNA expression was determined by quantitative real-time PCR and compared between CD55-silenced CSCs and NT control CSCs. Actin was used as a control. (C) Immunoblots of cisplatin-resistant parental cells silenced for CD55 using two shRNA constructs and a nontargeting control for CD55, NANOG, SOX2, and OCT4. Actin was used as a loading control. (D) Limiting dilution analysis of CD55 NT control compared with CD55 sh1 and sh2 silencing constructs in cisplatin-resistant parental cells. (E) Cell lysates from CD59-silenced A2780 CSCs and their NT controls were immunoblotted and probed with CD59, NANOG, SOX2, and OCT4. Actin was used as loading control. (F) Limiting dilution analysis plots of CD59 NT control compared with CD59 KD1 and KD2 silencing constructs in A2780 CSCs. *, $P < 0.05$; **, $P < 0.01$; ***, $P < 0.001$.

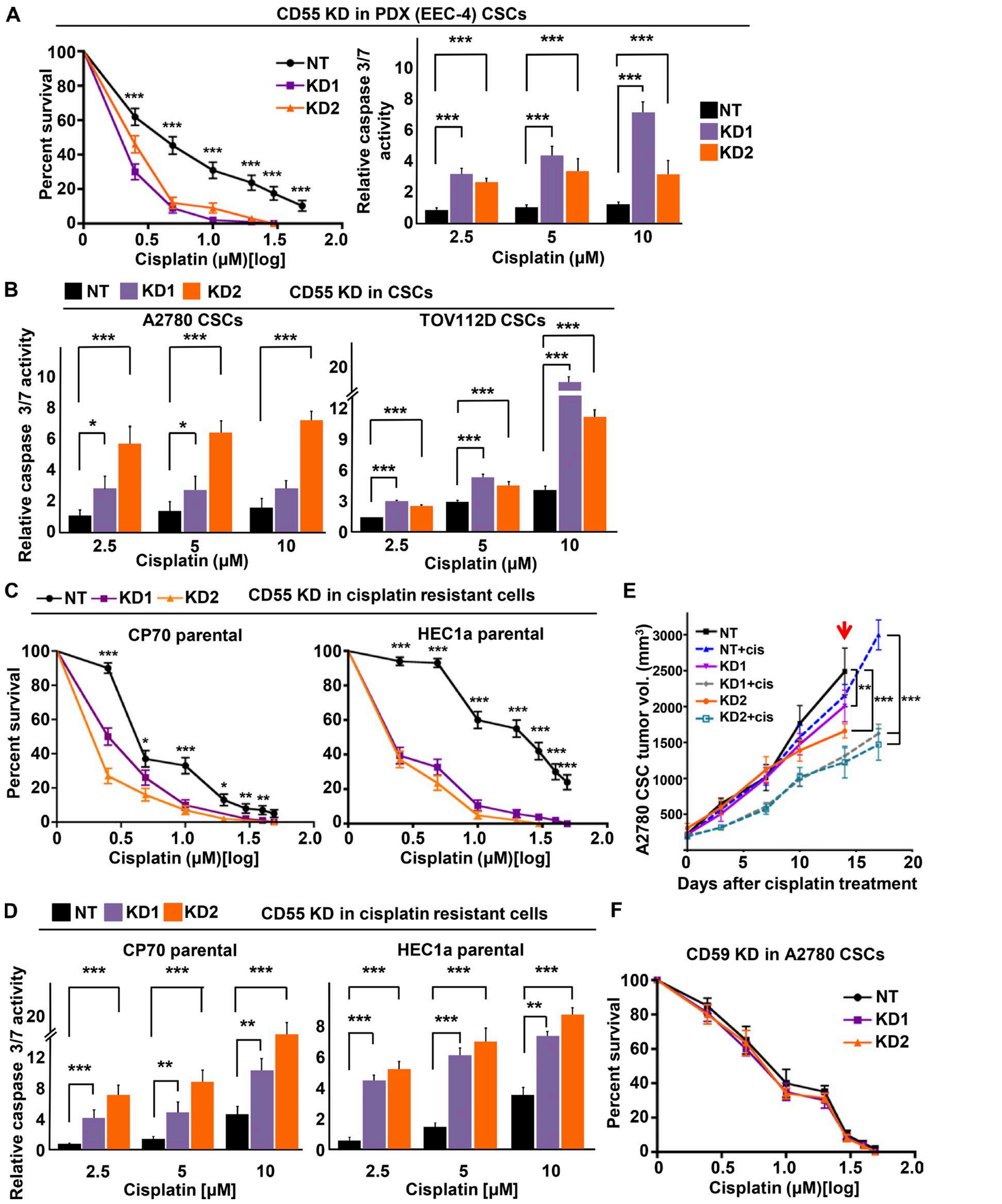

Figure S3. **CD55 maintains platinum resistance in patient-derived xenograft and cisplatin-resistant endometrioid tumors.** (A) CD55-silenced cisplatin-naive uterine PDX CSCs and their nontargeted (NT) controls were treated with cisplatin, and percentage of surviving cells and relative caspase 3/7 activity was analyzed. (B) Relative caspase 3/7 activity for CD55-silenced cisplatin-naive cells and their NT controls after treatment with cisplatin. Data are representative of three independent experiments. (C and D) CD55 silenced cisplatin-resistant parental cells and their NT controls were treated with cisplatin, and percentage of surviving cells and relative caspase 3/7 activity was graphed. (E) In vivo cisplatin sensitivity studies were performed comparing the NT control and CD55-silenced group. Graph shows the growth rate of tumors compared with the first day of cisplatin treatment. (F) CD59-silenced A2780 CSCs and their NT controls were treated with 0–50 μM cisplatin, and percentage surviving cells are graphed. *, $P < 0.05$; **, $P < 0.01$; ***, $P < 0.001$.

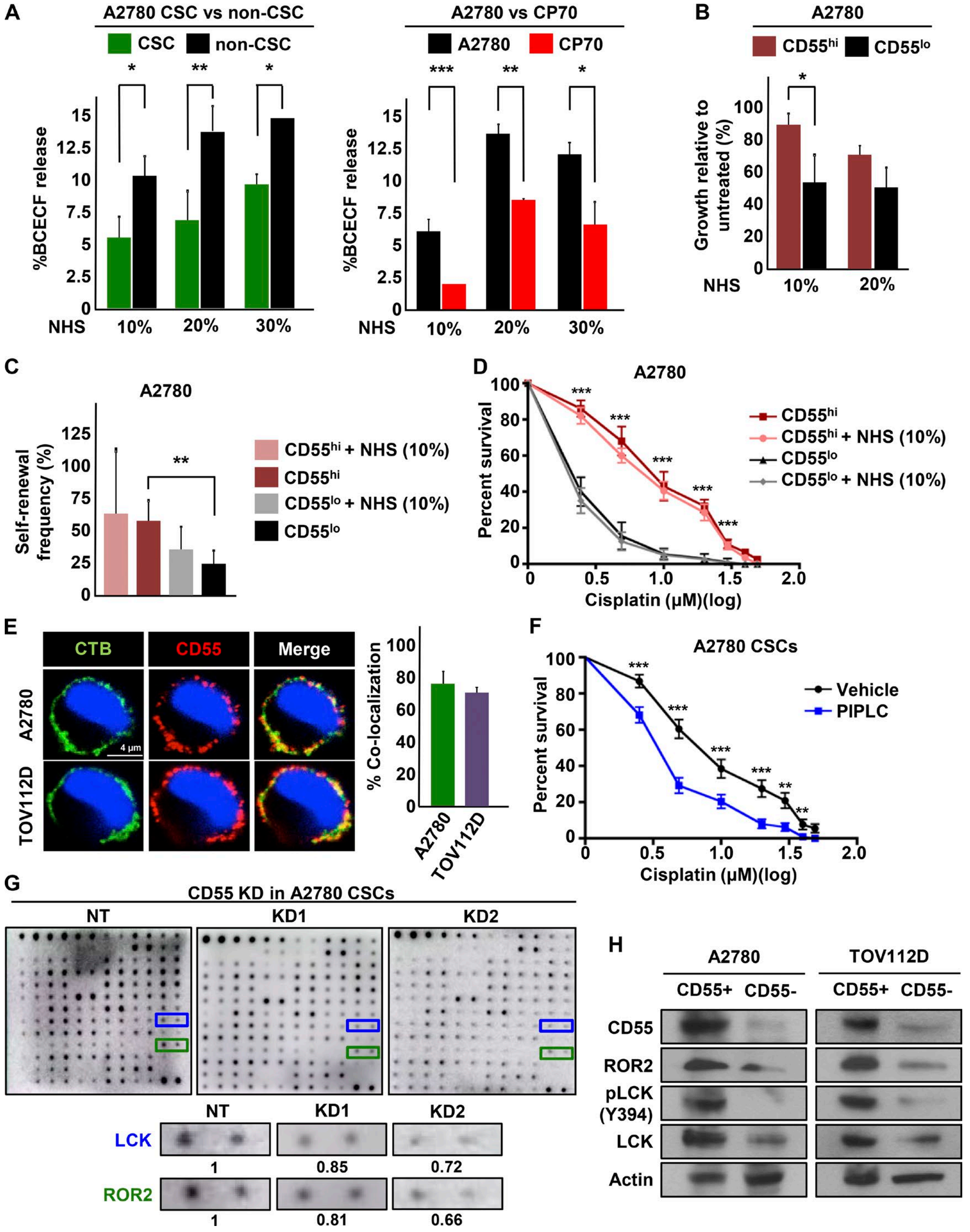

Figure S4. **CD55 regulates self-renewal and cisplatin resistance in a complement independent manner.** (A) Complement-mediated cytotoxicity was assessed by the percentage of BCECF dye release in CSCs versus non-CSCs and cisplatin-resistant versus cisplatin-naive cells treated with 10%, 20%, and 30% NHS. (B) A2780 cells sorted according to their surface CD55 expression were treated with 10% and 20% NHS, and growth relative to untreated controls was graphed. (C) Limiting dilution analysis plots of CD55+ and CD55− A2780 cells cultured with or without 10% NHS. (D) CD55+ and CD55− A2780 cells cultured with or without NHS were treated with 0–50 µM cisplatin, and percentage surviving cells was graphed. (E) Immunofluorescent staining of cisplatin-naive CSCs was performed for CD55 and cholera toxin B. (F) PIPLC-treated CSCs and their vehicle-treated controls were treated with 0–50 µM cisplatin, and percentage of surviving cells was graphed. (G) Receptor tyrosine kinase array was performed against 71 unique tyrosine kinases to identify the pathways altered by CD55 silencing in CSCs. (H) CSCs of cisplatin-naive cells were sorted according to surface CD55 expression and immunoblotted for CD55, ROR2, pLCK (Y394), and LCK. Actin was used as loading control. *, $P < 0.05$; **, $P < 0.01$; ***, $P < 0.001$. Bar, 4 µm.

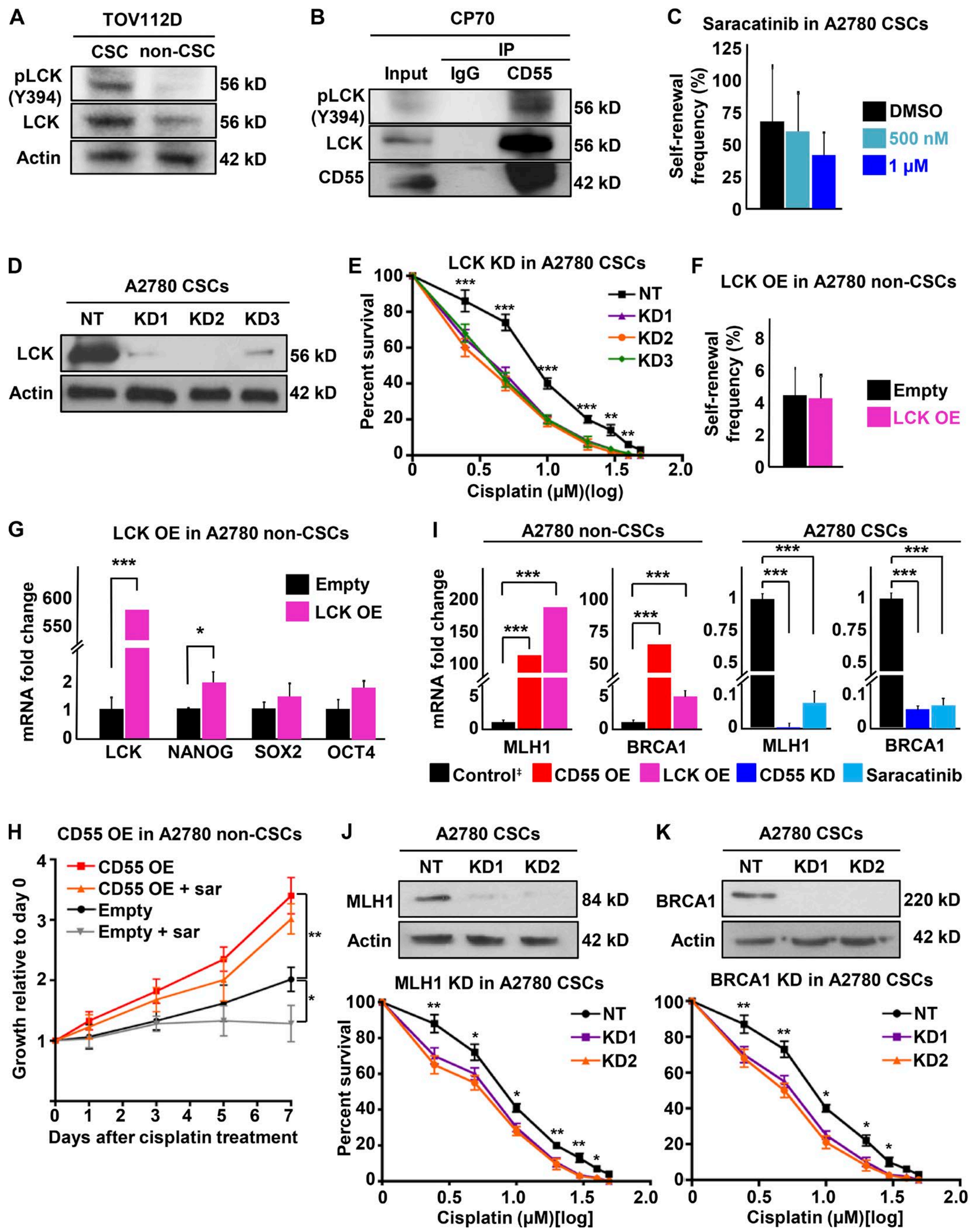

Figure S5. **CD55 signals via LCK and induces DNA repair genes.** (A) Cell lysates from TOV112D CSCs and non-CSCs were immunoblotted and probed with pLCK (Y394) and LCK. Actin was used as loading control. (B) Pull-down experiments with CD55 antibody were performed in CP70 parental cells, and elutes were probed for pLCK (Y394), LCK, and CD55. (C) Limiting dilution analysis plots of saracatinib and DMSO-treated cisplatin-naive CSCs. (D) Cell lysates from LCK-silenced A2780 CSCs and their nontargeted (NT) controls were immunoblotted and probed with LCK. Actin was used as loading control. (E) LCK-silenced CSCs and their NT controls were treated with 0–50 μM cisplatin, and percentage surviving cells is graphed. (F) Limiting dilution analysis plots of cisplatin-naive non-CSCs transduced with LCK overexpression and empty vector constructs. (G) mRNA expression was determined by quantitative real-time PCR and compared between LCK-overexpressing non-CSCs and empty vector control non-CSCs. Actin was used as a control. (H) Growth curves for CD55-overexpressing non-CSCs and their empty vector controls treated with or without saracatinib. The graph shows growth relative to day 0. (I) mRNA fold changes of the four most significantly modulated genes from gene expression profiling, comparing non-CSCs transduced with empty vector control versus CD55 or LCK overexpression, and CSCs with nontargeted control versus CD55 silencing, and CSCs with DMSO versus saracatinib treatment. (J and K) MLH1- and BRCA1-silenced CSCs and their nontargeted controls were treated with 0–50 μM cisplatin, and percentage surviving cells is graphed. ‡Empty vector control for non-CSCs and nontargeted control for CSCs. *, $P < 0.05$; **, $P < 0.01$; ***, $P < 0.001$.
